# *Dipylidium caninum* in the twenty-first century: epidemiological studies and reported cases in companion animals and humans

**DOI:** 10.1186/s13071-022-05243-5

**Published:** 2022-05-10

**Authors:** Julieta Rousseau, Andry Castro, Teresa Novo, Carla Maia

**Affiliations:** 1 Global Health and Tropical Medicine (GHTM), Instituto de Higiene e Medicina Tropical (IHMT), Universidade Nova de Lisboa (NOVA), Rua da Junqueira 100, 1349-008 Lisbon, Portugal; 2 Centre of Geographical Studies and Associated Laboratory (TERRA), Institute of Geography and Spatial Planning, Universidade de Lisboa, Edif. IGOT, Rua Branca Edmée Marques, 1600-276 Lisbon, Portugal

**Keywords:** *Dipylidium caninum*, Dogs, Cats, Humans, Siphonaptera, Epidemiology, Diagnosis, Treatment, Prevention, Zoonosis

## Abstract

**Background:**

Dipilidiosis is a parasitic disease caused by the tapeworm *Dipylidium caninum*. Fleas and, less frequently, lice act as an intermediate host, and their ingestion is required for infection to occur. While the disease mainly affects domestic and wild carnivores, it is also considered a zoonotic disease, with most human cases reported in children. *Dipylidium caninum* is considered to be the most common tapeworm infesting companion animals, but dipilidosis in humans is rare. The aims of this review were to improve current understanding of the epidemiology of this parasitosis and its management by the medical and veterinary community.

**Methods:**

A comprehensive review of the published literature during the last 21 years (2000–2021) on the epidemiology, clinical features, diagnosis, treatment and prevention measures of *D. caninum* infection and dipilidiosis in companion animals and humans was conducted.

**Results:**

Using predefined eligibility criteria for a search of the published literature, we retrieved and screened 280 publications. Of these, 161 (141 epidemiological studies, 20 case reports [16 human cases]) were considered for inclusion in this review. This parasitosis is present worldwide; however, despite being the most frequent cestode infection in animals, it is often underdiagnosed using common coprological techniques. Its diagnosis in humans has also proved challenging, being frequently confused with pinworm infection, leading to inappropriate treatment and to the persistence of the disease over time. Prevention measures include control of ectoparasites in animals and the environment, as well as regular deworming of animals, most commonly with praziquantel.

**Conclusions:**

The diagnosis of dipilidiosis remains challenging in both animals and humans, primarily due to the low sensitivity of the diagnostic methods currently available and a lack of knowledge of the morphological characteristics of the parasite. Although treatment with the appropriate anti-cestode compounds is well tolerated and results in resolution of the infection, indiscriminate use of these compounds may predispose to an increase in resistance. Given the worldwide distribution of this parasite, it is essential to act on several fronts, with a focus on health education for children and animal owners and the control of intermediate hosts, both in animals and in the surrounding environment.

**Graphical Abstract:**

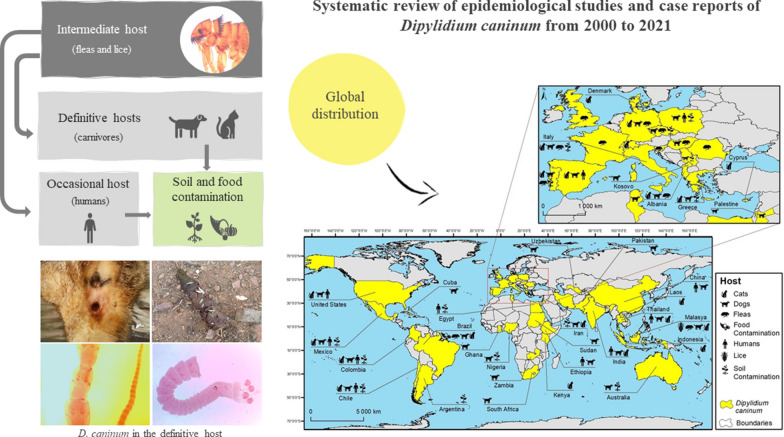

**Supplementary Information:**

The online version contains supplementary material available at 10.1186/s13071-022-05243-5.

## Background

Dipilidiosis is an underrated disease caused by the cestode *Dipylidium caninum*. The transmission of this parasite is complex as it involves an intermediate invertebrate host (flea or louse), which subsequently needs to be ingested by the definitive hosts, normally carnivores, but occasionally humans, for the infection to develop [1, 2]. In both cases, the infection is asymptomatic or the clinical signs or symptoms are non-specific and, consequently, proper diagnosis, treatment and prevention are challenging.

Climate change, coupled with increased urbanisation and the increased number of pets, both those with close relationships with their owners and also of sheltered or stray animals, may affect the prevalence and endemicity of the intermediate hosts [3–5]. If there is no effective ectoparasite control, the prevalence of pathogens they transmit, such as *D. caninum*, may also increase. This fact underlines the importance of raising awareness among the medical community, and the population in general, of the need for greater clinical suspicion of this cestode, as well as of the appropriate methods of diagnosis, treatment and prevention.

The aim of the present study was to summarise and analyse the epidemiology, pathogenesis, diagnosis and control measures of *D. caninum* infection and dipilidiosis in companion animals and humans, through a comprehensive review of the literature in the last 21 years (2000–2021), in order to raise awareness of the medical and veterinary community on the challenges associated with the management of this parasitosis.

## Search strategy, eligibility, and review

An online search was conducted of the MEDLINE® database on 10 November 2021 using the PubMed® (https://pubmed.ncbi.nlm.nih.gov/) search tool. The following search terms were used: ("Dipylidium" [MeSH Terms] OR "Dipylidium" [All Fields] OR “Dipilidi*” [MeSH Terms] OR “Dipilidi*” [All Fields]) AND ("dogs" [MeSH Terms] OR "dogs" [All Fields] OR "dog" [All Fields] OR "cani*" [All Fields] OR "cani*" [MeSH Terms] OR "cats" [MeSH Terms] OR "cats" [All Fields] OR "cat" [All Fields] OR "feli*" [All Fields] OR "feli*" [MeSH Terms] OR "human*" [MeSH Terms] OR "human*" [All Fields] OR "child*" [MeSH Terms] OR "child*" [All Fields] OR "people" [All Fields]). The search results were then filtered for the period 2000 to the present (10 November 2021) and extracted into a database in Microsoft Excel® (Microsoft Corp., Redmond, WA, USA) under a comma-separated-value (CSV) format.

**Fig. 1:**
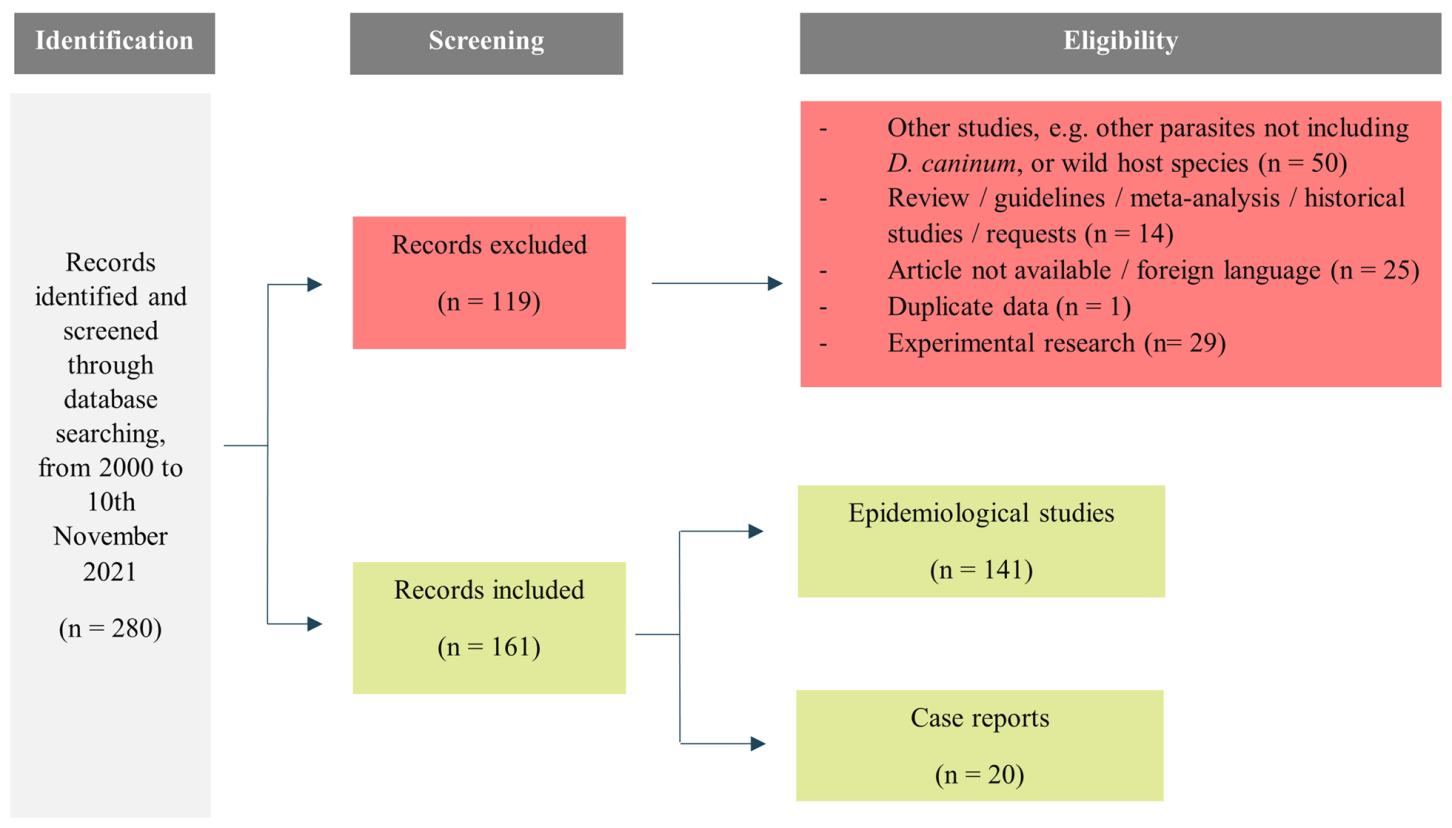
Schematic overview of the screening and selection process of the studies included in this review

All records were screened according to the title and abstract, if available. Two types of research were included: (i) epidemiological studies of *D. caninum* in dogs, cats, humans, fleas, lice and soil or food contamination studies; (ii) reported cases of dipilidiosis in dogs, cats and humans. Rejection criteria were: (i) studies of other parasites, i.e. not including *D. caninum*, or studies of *D. caninum* but in animal species other than dog or cat (e.g. wild hosts); (ii) review articles, guidelines, meta-analyses, historical studies and requests; (iii) unavailable articles or written in languages other than English, Spanish and Portuguese; (iv) duplicate studies; and (v) experimental studies. The study selection process is shown in Fig. 1.

When coprological methods were mentioned in the articles included in this review (Additional file 1: Table S1; Additional file 1: Table S2), we refer to them in a generic manner as one or more of the following: faecal smear, flotation and sedimentation. The reason for this is that different solutions, concentrations and protocols were used by the authors of the various studies, and the inclusion of such details would have made the overview of Tables S1 and S2 difficult.

## Parasite characteristics

### Aetiology and life-cycle

The helminth *D. caninum* is a cestode belonging to the order Cyclophyllidea and family Dipylidiidae. The biological cycle of this parasite is heteroxenous, occurring in the definitive host (carnivores and occasionally in humans) and intermediate host (fleas [*Ctenocephalides* spp. and *Pulex irritans*] and chewing lice [*Trichodectes canis* and *Felicola subrostratus*]) [6–9]. Carnivores, both domestic (dog [*Canis lupus familiaris*] and cat [*Felis catus*]) and wild, are the typical definitive hosts. In the latter, the parasite has been identified in several sylvatic species, namely foxes, wolves, jackals, hyaenas, coyotes, racoon dogs and cheetahs [10–12]. Transmission of and infestation by this parasite in both directions (wild to domestic and domestic to wild) is possible due to shared habitats, particularly at night, when wild animals come close to human populations in their forage for food [11, 12].

The infective larval form corresponds to a cysticercoid, which develops in the body cavity of the intermediate host [13]. The definitive host becomes infected through the ingestion of an infected flea or louse. In the small intestine of the mammalian host, the cysticercoid larva is digested and becomes fixed to intestinal wall by the scolex, initiating the adult developmental process. Wthin 2–3 weeks (prepatent period), the ovigerous proglottids detach from the strobilus and pass into the faeces [1, 2, 6, 13]. When the proglottids disintegrate, the larval stages of the intermediate hosts ingest the ovigerous capsules. The hexacanth embryos hatch and develop into cysticercoids in parallel with the invertebrate development [2, 6, 14].

### Morphology

Similar to other cestodes, *D. caninum* consists of a chain (strobilus) of segments (proglottids) that are independent of each other, with maturation progressing along the chain. Macroscopically, the adult parasite is a whitish flat worm ranging in length from 10 to 70 cm. The scolex is the narrower part of the parasite (diameter: < 0.5 mm) and is responsible for fixation of the parasite to the intestinal wall [1, 2, 6]. This attachment is possible due to its protruding and retractable rostellum, which bears three to four rows of hooks in the shape of a thorn, as well as four suckers [1, 2, 6, 13]. As the parasite matures, the proglottids become larger (size: 12 × 3 mm) and have mature genital organs. The seed-shaped ovigerous proglottids are loaded with eggs and ready to detach from the strobilus [6]. The eggs contain the first larval stage, also known as hexacanth embryo, and are grouped in thin-shelled capsules (size: 200 × 400 µm), with each containing five to 30 eggs (size: 40 × 50 µm [2, 6, 13, 15]. This tapeworm differs from other cestodes by having double genital pores, located slightly behind the middle of the lateral margins of each proglottid, and typical ovigerous capsules [2, 6, 13].

Due to the shape of the adult *D. caninum* and its biological characteristics, it is also known as the flea tapeworm, cucumber tapeworm and/or double-pored tapeworm [6].

## Epidemiology, prevalence, and distribution

**Fig. 2:**
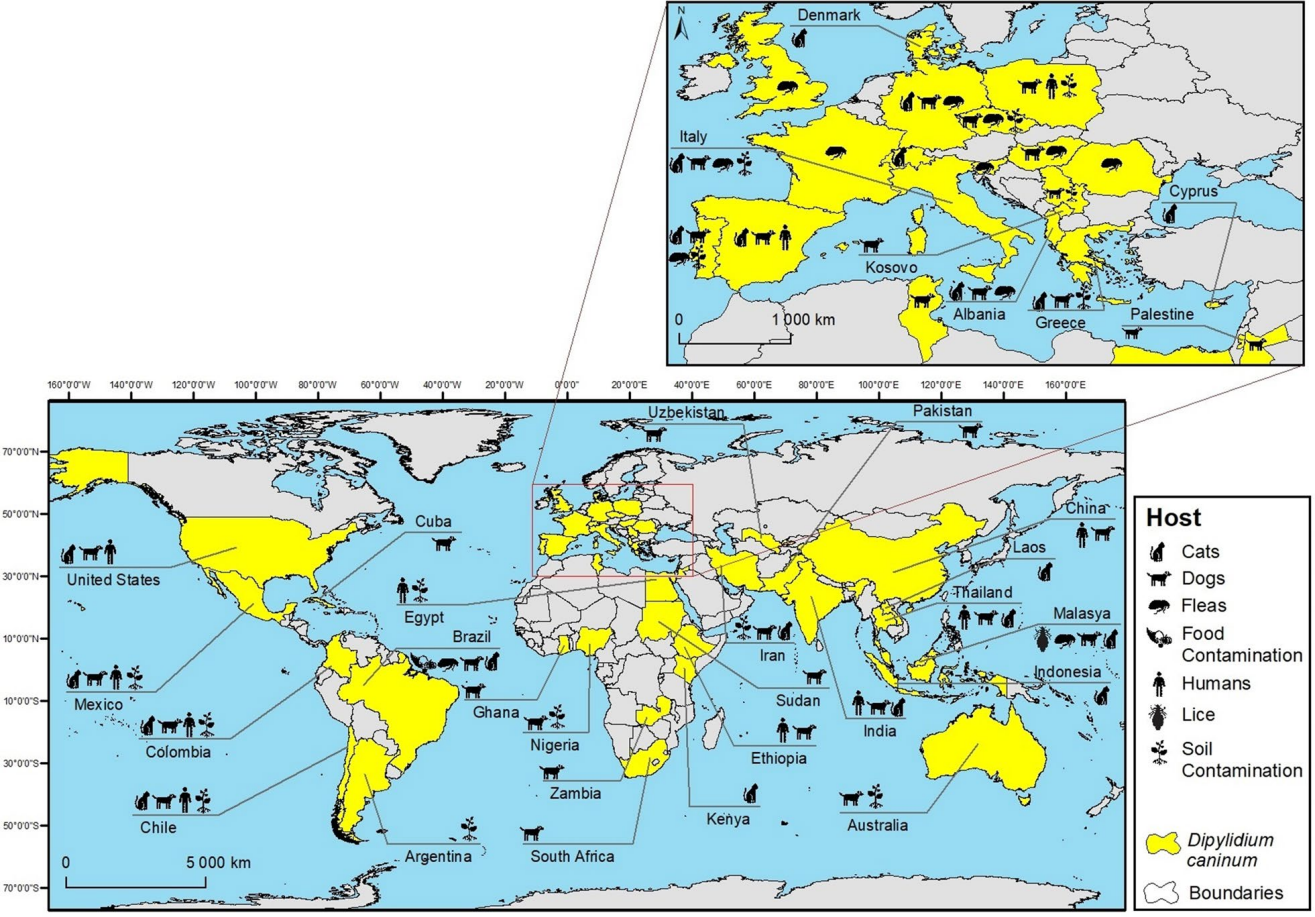
Global distribution of *Dipylidium caninum* between 2000 and 2021

*Dipylidium caninum* is distributed worldwide, occurring on all continents (with the exception of Antarctica) and detected either in vertebrates, including humans, and insects, namely fleas and lice [7, 16–21] (Fig. 2; Addotopma;file: Table S1). Several studies have shown soil contamination with this cestode (0.1–26.3%) [22–31], as well as food contamination (1.7%) [32]. The wide geographical distribution of this parasite is unsurprising, as invertebrate intermediate hosts are also found throughout the world, with fleas being the most frequent ectoparasite of dogs and cats [5, 21, 33]. The studies included in the present review were conducted in 50 countries, with most studies being from European (44/161, 27%), Asian (42/161, 26%) and North (27/161, 17%) and South (27/161, 17%) American countries (Fig. 2; Additional File 1: Table S1).

## Dogs and cats

Two distinct genotypes of *D. caninum* were have been identified in dogs and cats, suggesting the presence of two distinct species [34, 35]. In Spain, another species of the same genus, *Dipylidium carracidoi*, was also reported in necropsied cats, with a higher percentage of infection (32.8%) than reported for *D. caninum* (3%) [36]. According to the authors of this latter study, *D. carracidoi* is a relatively unknown species and there are very few reports on it; it seems to occur in this Spain and its life-cycle might be the same as that of *D. caninum* [36]. As the authors refer to the existence of morphological differences that allow the two species to be distinguished, it is possible that in some studies classification was incorrect, and that *D. carracidoi* was misidentified as *D. caninum*, and vice-versa [36].

The risk of *D. caninum* infection may vary depending on the vertebrate species and its lifestyle. Stray and shelter animals are less likely to have access to veterinary care and thus have a higher risk of infection [37–40]. Dogs and cats that are parasitised with fleas or lice have an increased risk of *D. caninum* infection [37]. Beugnet et al. [41] reported that in their study dogs had a higher percentage of fleas infected with the parasite. Regarding cats, their more pronounced grooming behaviour could lead to a higher flea intake compared to dogs and, consequently, a higher risk of *D. caninum* infection [41]. However, cats have been reported to show lower rates of *D. caninum* parasitism [16].

Few studies have assessed *D. caninum* prevalence according to the age of the animal, and the results of these studies are discordant. Some studies report a higher prevalence of the disease in young individuals [39, 42–44], which might be related with a protective immunity in older individuals [10], and others report higher prevalence with increasing age of the animals [1, 17, 45–50], which suggests a lack of post-infection protection [49]. Prevalence has also been reported to be associated with the animal's body temperature [14, 50]. Younger animals may have more difficulty in maintaining their body temperature, which impairs the development of cysticercoids inside the fleas [14, 50]. It has been suggested that differences in prevalence between sexes are more related to the social characteristics of the animals than to the sex itself [1, 17, 50, 51], as increased contact with other animals might be a risk factor for *D. caninum* infection [1, 51, 52].

Higher prevalence in rural or suburban areas (1.3–13.1%), compared to urban areas (0.7–5.7%), may be related to environmental conditions and a lower control of ecto- and endoparasites due to poorer veterinary care hampered by a greater distance to veterinary clinics/hospitals [53–57].

### Humans

Although humans are accidental hosts, children seem to be the most vulnerable to infection with *D. caninum* [21, 58] (Additional file 1: Table S2). This increased vulnerability of children is probably related to their close contact with animals, not only domestic animals but also stray animals and those without any veterinary care, as well as their poor hygiene habits, such as infrequent hand washing and playing and eating on the floor.

Adults may also become infected, with factors such as an immunosuppressive condition, bad hygiene habits and contact with animals without veterinary care being contributing factors [21, 59–61]. Contact with animals, either household animals or those outside the home, is considered a risk factor for infection [21, 62–64]. However, when there is no contact with animals, other means of transmission cannot be discarded, including the role of other ectoparasite vectors, such as the human flea (*P. irritans*) [9]; in addition, consideration must be given to immunosuppressive conditions or poor hygiene, both of which may facilitate infection [13, 59, 65]. The presence of proglottids or ovigerous capsules in soil or food [25, 28, 32, 58], as well as co-habitation with other infected people or companion animals [60], only represents an indirect risk of infection as the life-cycle of *D. caninum* is heteroxenous, and the infective cysticercoid larvae are only present in the intermediate host [2, 6].

## Clinical presentation

### Dogs and cats

The infection in dogs and cats is generally asymptomatic, even proglottids can be observed in the faeces [66–68]. However, a number of clinical signs are commonly associated with this parasitosis, such as anal pruritus, recognisable by scratching of the perineal region against a wall, as the ovigerous proglottids force/pass through the anal folds [67, 68]. This scratching behaviour is commonly known as scooting behaviour [67]. Other clinical signs that have been described include diarrhoea [67, 69], anorexia, weight loss [68, 69], dullness and poor hair coat [67]. Of note: there are often co-infections (Additional file 1: Table S1; Additional file 1: Table S2) with other gastrointestinal parasites, which may interfere with an understanding the true aetiology of the clinical signs [66, 69]. These co-infections may be particularly relevant not only for the clinical condition and synergic effects, which might lead to death [66, 69], but also due to their zoonotic potential, such as infections with *Ancylostoma* spp., *Toxocara* spp. and *Echinococcus* spp. [43, 48, 49, 51, 52, 57, 70–84].

### Humans

In humans, as in animals, infection with *D. caninum* can be asymptomatic [21, 58, 65, 85, 86], or non-specific symptoms may be observed, such as abdominal pain and discomfort [58, 59, 61, 62, 64, 87–89], bloating [64, 90], diarrhoea [17, 58, 59, 64, 90, 91], difficulty in defecation [60], anal itching [62–64, 92] that may lead to scratching of the perianal area and the development of abrasions and dermatitis [92], loss of appetite and less weight gain [58, 87] and occasional vomiting [17, 61] and fever [90]. Sleep disturbances, sadness, hyperactivity and irritability are also described [87, 88, 90, 92]. A few studies have reported haematological changes, namely leucocytosis [58], eosinophilia [58, 88], low haematocrit and/or haemoglobin [61, 64, 91], thrombocytopaenia, an increased erythrocyte sedimentation rate [61] and higher level of serum IgE [91]. It is also hypothesised that in humans it may be a self-limiting disease, with spontaneous cure [60]. In most of the clinical cases (Additional file 1: Table S2), proglottids were observed in the stool or perianal region, and described as grains of rice or as cucumber or other vegetable seeds, appearing individually or forming a chain [6, 21, 58, 60, 61, 63–65, 85–89, 91, 92]. In some cases, worm mobility was observed [65, 89, 92].

The detection of proglottids in the stool is one of the most frequent findings in infants and children due to caregivers observing the stool and the perianal region of children, particularly during diaper changes or bathing [60, 87, 89]. Since adults do not normally inspect their own stool, or at least not as often as they do their own children’s stool, more infections may go undetected in adults [59–61]. The lower prevalence of disease in adults is also likely to be related to their stronger immune system and fewer risk behaviours for acquiring the infection [58].

## Diagnosis

Traditional diagnosis is based on coprological methods, which are techniques that allow for the macroscopic and microscopic observation of parasites in the faeces. These methods are simple and inexpensive and can be performed in the setting of the veterinary surgery [15]. For the diagnosis of *D. caninum*, the coprological techniques performed are qualitative, such as faecal smear, flotation and sedimentation. The former, although fast, has the disadvantage of being not sensitive since the amount of stool analysed is very small and there is a lot of debris [15]. Flotation and sedimentation can be performed with different types of solutions and with or without a centrifugation step, ultimately the aim is to concentrate the parasitic elements present in a faecal sample to be observed under a microscope. With these techniques, diagnosis is based on the observation of ovigerous capsules, with five to 30 eggs [2]. Táparo et al. [93] evaluated the efficacy of different coprological methods for detecting the parasitic forms and found that sedimentation was the most efficient technique compared to faecal smear and flotation techniques. Possible explanations for these differences may be related to a higher specific gravity and the easy crystallisation of flotation solutions, leading to egg disintegration [94], as well as to the inability of egg capsules to float sufficiently due to their weight [6]. An additional disadvantage of coprology is that if the ovigerous capsule breaks, the eggs are indistinguishable from other taeniid eggs, possibly leading to an underestimation of *D. caninum* prevalence [15, 29].

In terms of other cestodes, quantitative techniques are of no value since the number of eggs found cannot be related to the number of adult parasites in the intestine and the excretion of proglottids occurs intermittently [13, 15, 95].

However, for the reasons described above, these traditional diagnostic methods generally present low sensitivity for *D. caninum*, which not only compromises diagnosis of the parasitosis but also leads to an underestimation of the real prevalence of the disease, as shown in the various epidemiological studies using these techniques [4, 43, 95, 96]. Studies based on coprological methods have obtained a prevalence ranging between zero and 39.1% [48, 97–101], whereas in necropsy-based studies the prevalence ranged between 0.9 and 83.3% [20, 102]. During necropsies, a more detailed analysis is performed, making this method more sensitive and reliable when compared with coprological techniques, as the adult parasites are observed in the small intestine of the animals [19, 29, 49, 77, 78, 82, 94, 103–110]. Therefore, epidemiological studies on animals based on necropsies will provide a more realistic insight into the prevalence of this cestode in the general population [94, 111–114]. In live animals, epidemiological studies are equally relevant, and to compensate for the lack of sensitivity of coprological methods, one or more of the following approaches can be adopted: increase sample size, apply molecular diagnostic methods and repeat the sampling of the same individuals over time [110]. In an individual diagnosis, and to increase the chances of finding proglottids or ovigerous capsules, the collection of fresh faeces for the coprological examination should preferably be done on 3 consecutive days, both in humans and other animals [37, 58, 94].

A detailed observation of the anal/perianal region and of faeces and/or gastrointestinal contents (during necropsies) is valuable for the detection of isolated proglottids or the strobilus of the cestode. After such samples have been recovered, the parasite can be identified by using appropriate stains (acid carmine) and further observation under the microscope or stereomicroscope [20, 81, 109, 115]. The typical features of *D. caninum* that will support the diagnosis include the presence of two sets of reproductive organs and double genital pores in the middle of each lateral edge, and, in ovigerous proglottids, the presence of ovigerous capsules [2, 6, 37, 65, 88].

Applying adhesive tape to the anal and perianal regions and subsequently observing what is attached to the tape under a microscope may be another diagnostic method. This procedure is extremely easy and inexpensive, but it should not be used exclusively, but rather as a complementary method to other methods, as its efficacy is debatable [52, 94, 101]. When performed during animal necropsies, if the perianal area is contaminated with fluid from the anal sacs, the eggs will not adhere properly to the adhesive [101].

More recently, molecular methods have been used for species identification of taeniid eggs [116], detection of cestode infection [10, 117] and in genetic studies of *Dipylidium* spp. [34, 35]. Zhu et al. [117] reported the simultaneous detection of *Taenia* sp. and *D. caninum* from dog faecal samples and adult parasites by a multiplex PCR assay using mitochondrial genes as molecular markers. The method stands out for its ability to discriminate and diagnose the different cestodes simultaneously, and in a single reaction, which makes the diagnosis faster and more sensitive. The sensitivity of this method may be increased if, before DNA extraction, the eggs are concentrated using a flotation or sedimentation technique [117].

In the studies included in this review, molecular methods were used for the detection of *D. caninum* in the intermediate hosts: fleas and lice [3, 7, 41] (Additional file 1: Table S1). Detection of the parasite’s genetic material in the invertebrate host represents only a potential infection, as it must be ingested by the vertebrate host [3]. However, this study emphasises the need to combine regular flea and lice control measures with tapeworm control measures [41]. Molecular approaches can also be used to identify potential new intermediate hosts capable of becoming infected and/or transmitting this tapeworm [3].

The presence of antibodies against *D. caninum* in serum can also be assessed by indirect haemagglutination (sensitivity of 73% and specificity of 90%) [1] or by specific enzyme-linked immunosorbent assays (sensitivity ranging from 50% to 100% and specificity ranging from 75% to 100%) [118]. The results of these tests indicate past and/or present infection, can guide diagnosis and treatment and can indicate the need for prevention [1]. However, the existence of cross-reactions, such as with Ancylostomatidae specimens, cannot be ruled out [118].

The anamnesis and a detailed clinical history can also be crucial to reaching the diagnosis of the disease. In animals, the presence of fleas or lice, and the infrequency of internal and external deworming, associated with clinical signs, may lead to the suspicion of *D. caninum* infection [67, 68, 80, 119, 120]. The presence of fleas or lice can be interpreted as a sign that sustains the infection by *D. caninum* since this parasitosis presupposes infestation by ectoparasites containing cysticercoid larvae [77, 80, 105, 108, 121]. However, the animal may no longer have fleas or lice, or the fleas or lice may not be detected at clinical examination [108, 121]. Also, the animal may have acquired the infection by contact with prey that were infested by infected arthropods [121].

As mentioned above, the clinical diagnosis of dipilidiosis can be challenging as the disease has a subclinical expression or the clinical signs and symptoms are predominantly non-specific, both of which preclude a diagnosis based on them [95]. Although the observation/report of rice-like worms in the faeces or in the anal, perianal, and tail regions (animals [81]) or in the stool, underwear, diapers and bath water (in humans) may be quite relevant, the intermittent elimination of proglottids and misdiagnosis, especially in humans [61, 87], often means that laboratory techniques are required to confirm the diagnosis.

In humans, contact with domestic or stray animals, frequent playing on the street or in playgrounds (especially children), immunosuppressive conditions and signs indicative of poor hygiene can also support the diagnosis [21, 59, 62–65, 122].

Proglottids have a physical resemblance to rice grains when dried, and to cucumber, pumpkin or watermelon seeds when humid; this may result in the proglottids being mistaken for vegetable matter, undigested food, maggots or fly larvae [6, 21, 65, 86, 88]. However, in humans, the most common misdiagnosis is that of the oxyurid nematode *Enterobius vermicularis* (pinworm), which causes symptoms identical to those caused by *D. caninum* and whose macroscopic appearance resembles that of *D. caninum* proglottids [21, 85, 88, 89, 92]. It is therefore important to distinguish between these two parasites, as different therapeutic options and prevention measures are required. Despite both moving actively up to the anus, they differ slightly in size (ovigerous proglottids of *D. caninum*: 2–3 mm; *E. vermicularis* specimens: 0.3–0.5 mm), and *D. caninum* ovigerous proglottids make an expanding and contracting movement along their length and have a flattened dorso-ventrally barrel shape compared to *E. vermicularis* that moves like a serpent and has a cylindrical shape [15, 58, 89].

Other differential diagnoses should also be taken into consideration, namely infection with other cestodes that can infect humans, such as *Hymenolepis* spp., *Taenia solium*, *T. saginata* and *Railletina* spp. [58, 85]. The correct diagnosis of each species can be achieved by a rigorous microscopic examination of the proglottids or by using molecular techniques [58, 85, 123]. In humans, *D. caninum* infection is considered to be rare, which may be related to the few symptoms it causes and the lack of knowledge about the disease, with consequent misdiagnosis. These factors possibly lead to disease underdiagnosis and underreporting [1, 58].

In companion animals, other differential diagnoses should be considered and may include bacterial, viral, fungal or parasitic infections, or other gastrointestinal diseases. Although scooting behaviour due to anal itching is characteristic of tapeworm infection, other conditions should also be discarded: anal sac disorders or allergic conditions, such as atopic dermatitis, flea bite allergic dermatitis or adverse food reaction [2, 67, 124].

## Treatment

### Dogs and cats

Although *D. caninum* infection is not very pathogenic in animals, with few clinical signs, the infection should be treated, especially due to its zoonotic potential [2, 37].

The drug of choice for the treatment of *D. caninum* infection in dogs and cats is praziquantel, administered either orally or subcutaneously at a single dose of 5 mg/kg [2, 8, 37, 67]. Other effective therapeutic options include epsiprantel at 2.75 mg/kg in cats and 5.5 mg/kg in dogs, and nitroscanate in dogs at a single dose of 50 mg/kg [2, 8, 37]. Despite praziquantel and epsiprantel being very effective, resistance to these two drugs has recently been reported in dogs infected with *D. caninum*, raising some concern, particularly as there are relatively few effective molecules to treat cestode infections in animals and humans [125]. If the anthelmintic used, either prophylactically or therapeutically, has no action against tapeworms, the animals will remain untreated for *D. caninum*, which not only delays diagnosis but also prolongs the disease and increases the risk to other animals and people. In addition, and as re-infection may occur, treatment with an anti-cestode should be combined with flea and lice control measures [2, 13].

Telluric fungi and bacteria can be used in biological control measures against parasites. When administered to animals, they are subsequently excreted in the faeces, thereby acting on the environment by eliminating possible immature stages of helminths present therein [13, 126–129]. Their use has been consistently reported in the control of parasitosis in large animals [126, 127]. This form of treatment could also be used in companion animals, particularly as a means to control *D. caninum* environmental contamination [13], as demonstrated by the in vitro studies with the nematophagous fungus *Pochonia chlamydosporia* [129], and with the bacterium *Bacillus thuringiensis* [128].

### Humans

The drug of choice to treat dipilidiosis in humans is also praziquantel [58, 65], at an oral single dose of 400–600 mg in adults and 10–20 mg/kg body weight in children [16, 21, 64, 65, 87, 92]. In heavy or persistent infections, a second dose may be necessary, administered at an interval of 2 to 4 weeks [16, 58]. There have also been reports of cases treated with higher doses (25 mg/kg) [85, 91], with multiple doses [61] or with a combination of different drugs (i.e. praziquantel + niclosamide) [58]. The reported decrease in the effectiveness of the recommended dosage may be related to the indiscriminate use of praziquantel in veterinary medicine, leading to the development of tolerance or resistance to the drug, or to cases of reinfection [58, 125]. Praziquantel is well-tolerated, but its use is not advised in pregnant or breastfeeding women [16, 58, 65].

Treatment with niclosamide, although effective, is more laborious as it requires prior preparation of the intestine with a liquid-based diet beginning in the afternoon of the day preceding treatment [16, 59, 60, 65].

The misdiagnosis of pinworm leads to the prescription of benzimidazoles, such as albendazole and mebendazole, or pyrantel and levamisole [21, 85, 88, 89, 92]. These molecules have no effect against tapeworms, and the incorrect diagnosis and subsequent non-effective treatment have resulted in a prolongation of the disease over time, which can vary from 1 month to 1 year [21, 61, 64, 86, 87, 89, 91, 92].

**Fig. 3:**
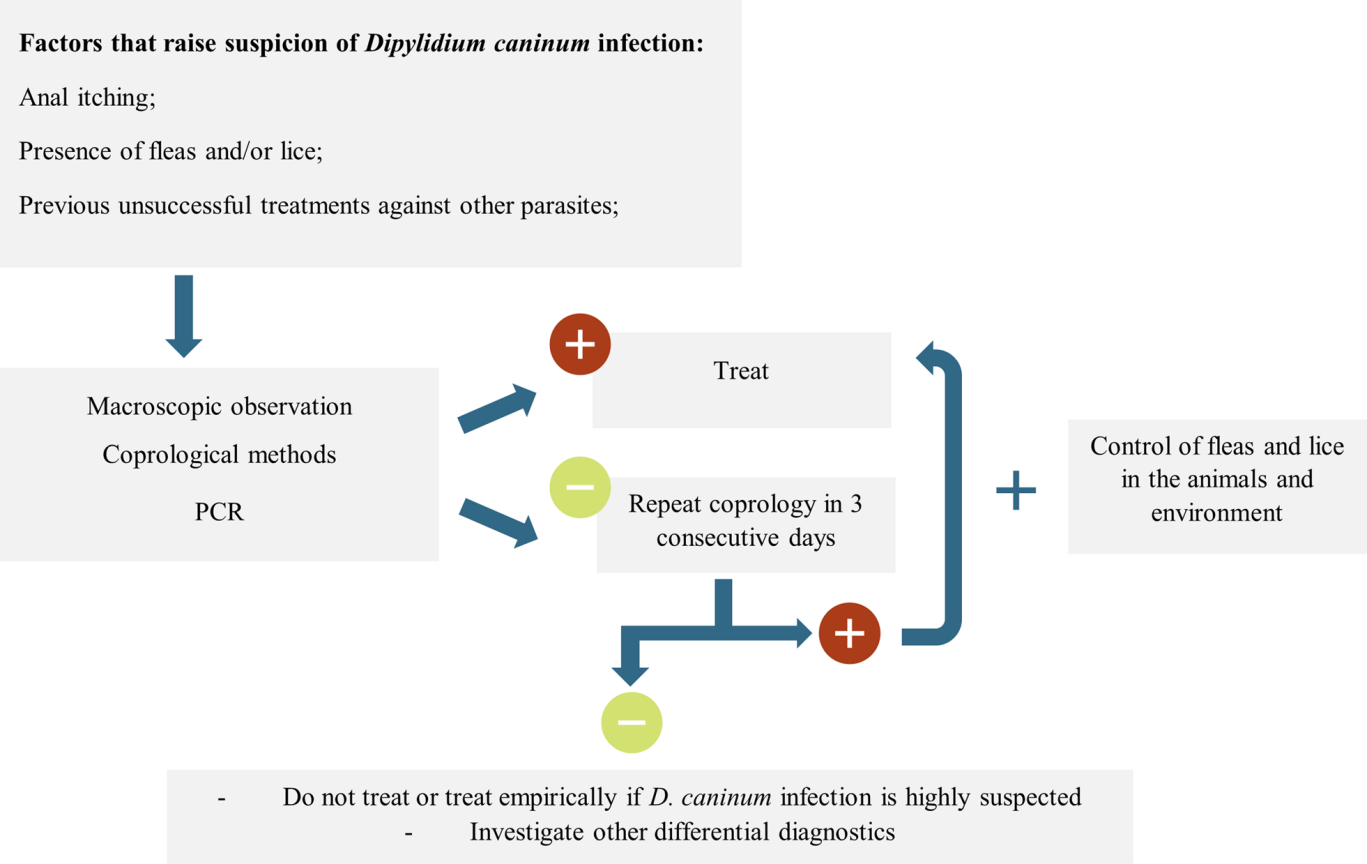
A schematic overview of the diagnostic and treatment procedures in animals and humans with suspected *D. caninum* infection

One possible approach in the diagnosis and treatment of *D. caninum* in animals and humans is schematised in Fig. 3.

## Preventive measures

In animals, a multi-pronged approach to control and prevent *D. caninum* is required. Most importantly, it is necessary to act at the level of the intermediary host. Light infestations of fleas or lice can easily go unnoticed, so a careful examination of the animal should be made at regular intervals with an appropriate comb, with the aim to detect these ectoparasites and/or their eggs and faeces in the animal's fur [130]. The administration of ectoparasiticides to all animals in the household all year round is also advised [85, 88, 130–132]. In addition to treatment, suggestions include the regular cleaning and vacuuming of the animal's resting areas, the proper cleaning of grooming utensils and the application, to the animal and/or in the animal’s environment, of insect growth regulators which, by acting on the immature forms, accelerate flea elimination [130]. Other preventive measures include coprological examinations once or twice a year to detect any infection, or when there is symptomatology that justifies it, and the prescription of an anthelminthic drug against tapeworms [37, 85, 131]. A veterinary examination, in combination with the recommended use of ectoparasiticides and anthelmintics throughout the year, can have a major impact on the prevalence of parasites in companion animals [133].

We highlight the fact that sometimes the lack of communication between physicians and veterinarians, or the omission of important parts of the clinical history and lifestyle, may lead to a missed or delayed diagnosis. In some human case reports [63, 88, 89], household pets were appropriately diagnosed and treated for *D. caninum* 2–3 months before symptoms appeared in the child; however, due to lack of communication or knowledge of the zoonotic capacity of this parasite, infants were diagnosed on more than one occasion with pinworm infection and treated with mebendazole, which did not cure the tapeworm infection. Both the medical and veterinary community should therefore raise awareness of zoonotic diseases and their prevention measures, with education of sanitation and hygienic measures being a priority [134]. Physicians should also ask about contact with animals inside and outside the house and whether these animals have recently presented any similar clinical signs [21].

The detection of *D. caninum* in children's playgrounds emphasises the need for greater protection of these places against the entry of animals, as well as the importance of removing animal excrements from public areas and thus preventing soil contamination [26, 58, 85, 122, 135]. Children should be advised to avoid touching or playing with stray animals as they are usually poorly protected against parasites [21, 85, 88]. In addition, children should wash their hands frequently, particularly after playing on the floor or with animals, and should not eat on the floor, as contamination of the food with the intermediate hosts may occur [21, 26]. Humans should avoid being licked by animals [88] as their saliva may be contaminated with the cysticercoid larva [88, 89, 136, 137]. In one study from Brazil, *D. caninum* was present in one vegetable from a supermarket (1.7%, 1/60). Although *D. caninum* eggs present in soil or food are not the infective form of the parasite, their detection in these types of samples reveals human or animal faecal contamination and highlights, once again, the importance of good hygiene practices, particularly during food preparation and consumption [32].

## Conclusions

*Dipylidium caninum* has been detected worldwide, which is a consequence of the global distribution of its intermediate hosts. Its infection has complex characteristics in terms of transmission, clinical signs, diagnosis, treatment and prevention. Only with a comprehensive knowledge of these characteristics can the clinical suspicion of this parasitosis in animals and humans be increased, appropriate treatments and effective preventive measures implemented and a greater sanitary education secured. It is therefore essential to alert the medical and veterinary community to this zoonotic parasite, which has been underrated, but which may become more frequent in the future.

## Supplementary Information

**Additional file 1: Table S1.** Epidemiological studies of *Dipylidium caninum* in dogs, cats, human, fleas, louses and soil and food contamination (2000–2021). **Table S2.** Case reports of *Dipylidium caninum* in humans, dogs and cats (2000–2021).

## Data Availability

The references supporting the conclusions of this review are cited in the text, and data are also available in additional files.
