# Supplementary material for: Dipylidium caninum in the twenty-first century: epidemiological studies and reported cases in companion animals and humans
Source: Parasit Vectors. 2022 May 10;15:131. doi: 10.1186/s13071-022-05243-5 (PMC9088078; doi:10.1186/s13071-022-05243-5)
Supplement: Supplementary file 1 — Additional file 1: Table S1. Epidemiological studies of Dipylidium caninum in dogs, cats, human, fleas, louses and soil and food contamination (2000–2021). Table S2. Case reports of Dipylidium caninum in humans, dogs and cats (2000–2021). [file 13071_2022_5243_MOESM1_ESM.docx]

**Table S1.** Epidemiological studies of *Dipylidium caninum* in dogs, cats, human, fleas, louses, and soil and food contamination (2000-2021)

| **Continent** | **Country** | **Sampling year** | **Host(s)** | **Lifestyle / origin / age** | **Sample (N)** | **Diagnostic method** | **Prevalence (% of positive samples)** | **Co-infection / infestation** | **Observations** | **Reference** |
| --- | --- | --- | --- | --- | --- | --- | --- | --- | --- | --- |
| **Africa** | **Egypt** | 2018 | Human | Children (1- 15 years old) | Faecal samples (996) | Macroscopic observation and coprological methods | 0.4% | nd | Half of these positive cases had gastrointestinal discomfort and anal pruritus | [1] |
|  |  | 2013-15 | Cats | Stray / soil: sandy spots, streets, markets) | Faecal samples (170) | Macroscopic observation and coprological methods | 18.82% | nd | na | [2] |
|  |  | 2010 | Cats | Stray / soil: sandy spots | Faecal samples (113) | Macroscopic observation and coprological methods | 5% | nd | na | [3] |
|  | **Ethiopia** | 2017-18 | Dogs;  Humans | Semi-stray  Children | Faecal samples (dogs: 384; humans: 259) | Macroscopic observation and coprological methods | Dogs – 21%; Children – 0.4% | nd | For children, there was 1 positive case: 3 years old child with nausea, vomiting and diarrhoea for 3 days | [4] |
|  | **Ghana** | nd | Dogs | Owned | Faecal samples (380) | Coprological methods | 13.4% | nd | na | [5] |
|  | **Kenya** | 2015 | Cats | Owned | Faecal samples (103) | Coprological methods | 8.7% | nd | na | [6] |
|  | **Nigeria** | 2018 | Dogs | Owned | Faecal samples (200) | Coprological methods | 4.5% | nd | na | [7] |
|  |  | 2007-08 | Dogs | Stray | Faecal samples (413) | Coprological methods | 7.5% | *Trichuris vulpis*; *Toxocara* spp.; *Ancylostoma caninum* | na | [8] |
|  |  | 2006- 07 | Dogs | Owned | Faecal samples (396) | Macroscopic observation and coprological methods | 9.1% | nd | na | [9] |
|  |  | 2005-06 | na | Playgrounds | Soil (608) | Sieving method (flotation-centrifugation) | 16.8% | nd | na | [10] |
|  |  | 2004 | Dogs | Sheltered  and stray | Faecal samples (269) | Coprological methods | 4.1% | nd | Prevalence for each lifestyle was not discriminated | [11] |
|  |  | 2001-02 | Dogs | Owned | Faecal samples (959) | Coprological methods | 0.2% | nd | na | [12] |
|  | **South Africa** | 1998-99 | Dogs | Stray | GI contents and faecal samples, adhesive tape (63) | Macroscopic and microscopic observation of parasites collected during necropsy,  coprological methods and adhesive tape in perianal skin and hair | 44.4% | nd | Prevalence for all methods combined | [13] |
|  |  | 1997-98 | Dogs | Owned | GI contents and faecal samples (69) | Macroscopic and microscopic observation of parasites collected during necropsy, and coprological methods | 39.1% | nd | na | [14] |
|  | **Sudan** | 2018 | Dogs | Stray | Faecal samples (360) | Coprological methods | 3.1% | nd | na | [15] |
|  | **Tunisia** | 2002-03 | Dogs | Stray | Faecal samples (375) | Macroscopic observation and coprological methods | 8.59% | nd | na | [16] |
|  | **Zambia** | 2010 | Dogs | Owned | Faecal samples (452) | Macroscopic observation and coprological methods | 6.2% | *A. caninum* | na | [17] |
|  |  | 2005-06 | Dogs | Owned | Faecal samples (540) | Coprological methods | 2.2% | nd | na | [18] |
| **Asia** | **China** | 2015 | Dogs with diarrhoea | Owned | Faecal samples (485) | Coprological methods | 0.2% | nd | na | [19] |
|  |  | 2006-07 | Dogs | Owned | GI contents (438) | Macroscopic and microscopic observation of parasites collected during necropsy | 42.3% | nd | na | [20] |
|  |  | 2002-03 | Dogs | Owned | Faecal samples (371) | Macroscopic and microscopic observation | 1.08% | *Echinococcus multilocularis*; *Taenia* spp. | na | [21] |
|  | **India** | 2010-11 | Dogs | Owned and stray | Faecal samples (owned:172, stray: 74) | Macroscopic observation and coprological methods | 17.39% | nd | Prevalence for each lifestyle was not discriminated | [22] |
|  |  | 2008-09 | Dogs | Owned | Faecal samples (200) | Macroscopic observation | 3.5% | *A. caninum* | na | [23] |
|  |  | 2005-09 | Cats | Stray | GI contents (27) | Macroscopic and microscopic observation of parasites collected during necropsy | 40.7% | nd | na | [24] |
|  | **Indonesia** | 2018-19 | Cats | Owned and stray | Faecal samples (owned: 60; stray: 60) | Coprological methods | Owned – 3.33%; Stray – 0% | *Ancylostoma* sp.; *Eimeria* sp. | na | [25] |
|  | **Iran** | 2017-18 | Dogs | nd / soil: livestock farming zones | Faecal samples (130) | Coprological methods | 25.5% | nd | na | [26] |
|  |  | 2017-18 | Dogs | Stray | GI contents (20) | Macroscopic and microscopic observation of parasites collected during necropsy | 45% | nd | na | [27] |
|  |  | 2016-18 | Dogs | Owned and stray | Faecal samples (owned: 144; stray: 408) | Coprological methods | Owned – 2.7%; Stray – 2,4% | nd | na | [28] |
|  |  | 2014-15 | Cats | Stray | GI contents (104) | Macroscopic and microscopic observation of parasites collected during necropsy | 23.1% | *Taenia taeniformis* | na | [29] |
|  |  | 2014-15 | Dogs | Owned and stray | Faecal samples (owned: 120; stray: 181) | Coprological methods | Owned – 4.2%; Stray – 3.3% | nd | na | [30] |
|  |  | 2013 | Cats | Stray | GI contents and faecal samples (51) | Macroscopic and microscopic observation of parasites collected during necropsy, and coprological methods | 29.41% | nd | na | [31] |
|  |  | 2013 | Cats | Stray | GI contents (50) | Macroscopic and microscopic observation of parasites collected during necropsy | 58% | nd | na | [32] |
|  |  | 2011-12 | Dogs | Stray | GI contents (100) | Macroscopic and microscopic observation of parasites collected during necropsy | 39% | nd | na | [33] |
|  |  | 2012 | Cats | Stray / soil: parks, squares, playgrounds | Faecal samples (140) | Coprological methods | 2.9% | nd | na | [34] |
|  |  | 2009-10 | Dogs | Owned and stray | Faecal samples (owned: 28; stray: 49) | Macroscopic observation and coprological methods | Owned – 4%; Stray – 0% | nd | na | [35] |
|  |  | 2009-10 | Cats | Stray | GI contents and faecal samples (52) | Macroscopic and microscopic observation of parasites collected during necropsy, and coprological methods | 23.07% | nd | na | [36] |
|  |  | 2009 | Dogs | Stray | GI contents (14) | Macroscopic and microscopic observation of parasites collected during necropsy | 7.2% | nd | na | [37] |
|  |  | 2008-09 | Dogs | Stray | GI contents and faecal samples (100) | Macroscopic and microscopic observation of parasites collected during necropsy, and coprological methods | 46% | nd | na | [38] |
|  |  | 2006-08 | Dogs | Owned | Faecal samples (59) | Coprological methods | 0% | nd | na | [39] |
|  |  | 2007 | Dogs | Stray | GI contents and faecal samples (50) | Macroscopic and microscopic observation of parasites collected during necropsy, and coprological methods | 36% | nd | na | [40] |
|  |  | 2006 | Dogs | Stray | GI contents and faecal samples (50) | Macroscopic and microscopic observation of parasites collected during necropsy, and coprological methods | 4% | nd | na | [41] |
|  |  | 2005-06 | Cats | Stray | GI contents (114) | Macroscopic and microscopic observation of parasites collected during necropsy | 45.6% | nd | na | [42] |
|  |  | 2004-05 | Cats | Stray | GI contents and faecal samples (113) | Macroscopic and microscopic observation of parasites collected during necropsy, and coprological methods | 68.1% | nd | na | [43] |
|  |  | nd | Dogs | Stray | GI contents (83) | Macroscopic and microscopic observation of parasites collected during necropsy | 38.55% | nd | na | [44] |
|  | **Jordan** | 1994-95 | Dogs | Stray | GI contents (94) | Macroscopic and microscopic observation of parasites collected during necropsy | 51.1% | *Echinococcus granulosus*; *Taenia* spp.; *Toxocara* sp.; *Toxascaris* sp.; *Mesocestoides* sp. | na | [45] |
|  | **Laos** | 1989 | Cats | Owned | GI contents (55) | Macroscopic and microscopic observation of parasites collected during necropsy | 24% | nd | na | [46] |
|  | **Malaysia** | 2011 | Dogs; Cats | Owned | Faecal samples (dogs: 77; cats: 28) | Coprological methods | Dogs – 3.9%; Cats – 7.1% | *Ancylostoma* spp.; *Toxocara* spp.; *Toxascaris leonina*; *Entamoeba*; *Giardia duodenalis* | na | [47] |
|  |  | 2007-10 | Cats | Stray | GI contents (543) | Macroscopic and microscopic observation of parasites collected during necropsy | 6.6% | nd | na | [48] |
|  |  | nd | Fleas and louse | na | Fleas and louse (92 *Ctenocephalides felis* and 30 *Felicola subrostratus* from 20 stray cats; and 26 *Ctenocephalides orientis* from 13 stray dogs) | PCR (28S rRNA, 12S rRNA) | *C. felis* – 2.2%*; F. subrostratus* – 10%*; C. orientis* – 0% | nd | na | [49] |
|  | **Palestine** | 2019 | Dogs | Owned and stray | Faecal samples (owned: 64; stray: 86) | Macroscopic observation and coprological methods | 23% | nd | Prevalence for each lifestyle was not discriminated | [50] |
|  | **Thailand** | 2014-15 | Cats | Owned | Faecal samples (509) | Coprological methods | 0.4% | nd | ba | [51] |
|  |  | nd | Dogs; Cats | Sheltered | Faecal samples (dogs: 500; cats: 300) | Coprological methods | Dogs – 0.2%; Cats – 0% | nd | na | [52] |
|  |  | nd | Dogs; Humans | Semi-domesticated/stray; nd | Faecal samples (dogs: 204; humans: 204) | Coprological methods | Dogs – 4.4%; Humans – 0% | nd | na | [53] |
|  | **Uzbekistan** | 2014-18 | Dogs | Owned and stray | Faecal samples (owned: 1749; stray: 5) | Coprological methods | 0.9% | nd | Prevalence for each lifestyle was not discriminated | [54] |
| **Europe** | **Various countries** (Albania, Bulgaria, Czech Republic, France, Germany, Hungary, Italy, Portugal, Romania, Slovenia) | 2009-13 | Fleas | na | Fleas (1696 *C. felis* from 435 cats; and 732 from 178 dogs. 2828 *C. canis* from 396 dogs) | PCR (28S rDNA) | *C. felis* from cats – 2.23%; *C. felis* from dogs – 5.2%;  *C. canis* from dogs – 3.1% | nd | na | [55] |
|  | **Albania** | 2008-09 | Cats | Owned | GI contents (18) | Macroscopic and microscopic observation of parasites collected during necropsy | 83.3% | nd | na | [56] |
|  |  | 2004-09 | Dogs | nd | GI contents (111) | Macroscopic and microscopic observation of parasites collected during necropsy | 65.8% | nd | na | [57] |
|  | **Cyprus** | nd | Cats | Owned and sheltered | Faecal samples (185) | Coprological methods | 0.5% | nd | Prevalence for each lifestyle was not discriminated | [58] |
|  | **Czech Republic** | 1998-2000 | Dogs | Urban (soil), rural (owned and soil), and sheltered | Faecal samples (urban areas: 3780; rural areas: 540; sheltered: 524) | Coprological methods | Urban – 0.7%; Rural – 1.3%; Sheltered – <2% | nd | na | [59] |
|  | **Denmark** | 2014 | Cats | Owned and stray | GI contents (owned: 7; stray: 92) | Macroscopic and microscopic observation of parasites collected during necropsy, and coprological methods | 1% | nd | Prevalence for each lifestyle was not discriminated | [60] |
|  | **Germany** | 2003-10 | Dogs; Cats | Owned | Faecal samples (dogs: 24677; cats: 8560) | Coprological methods | Dogs – <0.1%; Cats – <0.1% | nd | na | [61] |
|  |  | 1999-2002 | Dogs; Cats | nd | Faecal samples (dogs: 8438; cats: 3167) | Coprological methods | Dogs – 0.4%; Cats – 0.1% | nd | na | [62] |
|  | **Greece** | 2016 | Cats | Owned and stray | Faecal samples (1150) | Coprological methods | 0.2% | nd | Prevalence for each lifestyle was not discriminated | [63] |
|  |  | 2015-16 | Dogs | Owned and stray | Faecal samples (owned: 630; stray: 406) | Macroscopic observation and coprological methods | 3.4% | *Toxocara canis* | Prevalence for each lifestyle was not discriminated | [64] |
|  |  | 2015 | Cats | Stray | Faecal samples (150) | Coprological methods | 2% | nd | na | [65] |
|  |  | 2011-15 | Dogs; Cats | Owned and sheltered | Faecal samples (dogs: 879; cats: 264) | Coprological methods | Dogs – 0.2%; Cats – 0% | nd | Prevalence for each lifestyle was not discriminated | [66] |
|  |  | 2003-04 | Dogs | Owned | Faecal samples (281) | Coprological methods | 0.3% | nd | na | [67] |
|  | **Hungary** | nd | Dogs | Owned and sheltered | Faecal samples (490) | Coprological methods | 0.61% | nd | Prevalence for each lifestyle was not discriminated | [68] |
|  | **Italy** | 2015 | Dogs | Sheltered | Faecal samples (148) | Coprological methods | 0.7% | nd | na | [69] |
|  |  | 2012-14 | Cats | Owned, sheltered and stray | Faecal samples (owned: 140; sheltered: 64; stray: 63) | Coprological methods | 1.8% | *Tritrichomonas foetus* | Prevalence for each lifestyle was not discriminated | [70] |
|  |  | 2011-14 | Dogs | Sheltered | Faecal samples (639) | Macroscopic observation and coprological methods | 0.5% | nd | na | [71] |
|  |  | 2006-12 | Dogs | Owned and sheltered | Faecal samples (owned: 1156; sheltered: 1619) | Coprological methods | Owned – 0.1%; Sheltered – 0.1% | nd | na | [72] |
|  |  | 2010 | nd | Soil | Faecal samples (463) | Microscopic examination of proglottids from faeces; Coprological methods | 0.43% | nd | na | [73] |
|  |  | 2008-10 | Dogs; Cats | Owned | Faecal samples (dogs: 239; cats: 81) | Macroscopic observation and coprological methods | Dogs – 1.25%; Cats – 1.2% | Fleas | na | [74] |
|  | **Kosovo** | 2003-04 | Dogs | Owned and stray | Faecal samples (Owned: 136 pet dogs, 33 sheep dogs, 54 hunting dogs; Stray: 82) | Coprological methods | Owned: Pet – 2.2%; Sheep – 0%; Hunting – 0%. Stray: 6.1% | nd | na | [75] |
|  | **Poland** | 2017 | na | Sandpits and children play areas | Soil (29) | Flotation | 6.9% | nd | na | [76] |
|  |  | 2012-14 | Dogs | nd / soil: public places | Faecal samples (339) | Macroscopic observation and coprological methods | 5.2% | nd | na | [77] |
|  |  | nd | Dogs | nd | Faecal samples (763) | Macroscopic observation and coprological methods | 4.07% | nd | na | [78] |
|  | **Portugal** | 2019-20 | Dogs | Stray | Faecal samples (63) | Macroscopic observation and coprological methods | 6% | nd | na | [79] |
|  |  | 2011-2012 | Dogs | Farm and hunting dogs, and soil | Faecal samples (farm dogs: 195; hunting dogs: 101; soil: 296) | Microscopic examination of proglottids from faeces; Coprological methods | Farm dogs – 1.02%; Hunting dogs – 0.99%; Soil – 0.68%; | Ancylostomatidae | na | [80] |
|  |  | 2009-11 | Cats | Stray | GI contents and faecal samples (162) | Macroscopic and microscopic observation of parasites collected during necropsy, and coprological methods | 53.1% | nd | na | [81] |
|  |  | 2003-05 | Cats | Stray | Faecal samples (74) | Coprological methods | 1.4% | nd |  | [82] |
|  | **Serbia** | 2018 | nd | Soil: kindergartens, parks, public squares | Faecal samples (282) | Coprological methods | 27% | nd | na | [83] |
|  |  | 2017-18 | Dogs | Sheltered | Faecal samples (1267) | Macroscopic observation in faecal samples | 4.3% | Ancylostomatidae; Taenidae | na | [84] |
|  | **Spain** | 2008 | Cats | Stray | GI contents (58) | Macroscopic and microscopic observation of parasites collected during necropsy | *D. caninum* – 3%; *D. carracidoi* – 32.8% | nd | na | [85] |
|  |  | nd | Cats | Stray | GI contents and faecal samples (48) | Macroscopic and microscopic observation of parasites collected during necropsy, and coprological methods | 64.6% | nd | na | [86] |
|  |  | nd | Dogs | Owned, sheltered and stray | GI contents and faecal samples (owned: 125; sheltered: 100; stray: 50) | Macroscopic and microscopic observation of parasites collected during necropsy, and coprological methods | 39% (Coprology – 1%; Necropsy – 38%) | nd | Prevalence for each lifestyle was not discriminated | [87] |
|  |  | nd | Dogs | Sheltered | GI contents and faecal samples (1800) | Macroscopic and microscopic observation of parasites collected during necropsy (n=300), and coprological methods (n=1500) | 13.22%; (Coprology – 9.67-8%; Necropsy – 31%) | nd | na | [88] |
|  |  | nd | Dogs | Sheltered | Faecal samples (1161) | Coprological methods | 0.9% | nd | na | [89] |
|  |  | nd | Cats | Owned, farm and stray | Faecal samples (owned: 103; farm: 48; stray: 231) | Coprological methods | Owned – 0%; Farm – 2%; Stray – 3.9% | nd | na | [90] |
|  | **Switzerland** | 2012-15 | Cats | Owned, sheltered and stray | Faecal samples (owned: 299; sheltered: 197; stray: 168) | Macroscopic observation and coprological methods | Owned – 0%; Sheltered – 0%; Stray – 2.4% | nd | na | [91] |
|  | **United Kingdom** | 2018 | Fleas | na | Fleas (>90% *C. felis* from 662 and 812 owned dogs and cats, respectively) | PCR (28S rRNA) | 3% | nd | Fleas of the same species from each infested host were pooled (470 pooled flea samples) | [92] |
| **North America** | **Mexico** | 2017-18 | Dogs | Stray | GI contents (103) | Macroscopic and microscopic observation of parasites collected during necropsy, and coprological methods | 16.5% | *Taenia* spp. | na | [93] |
|  |  | 2013 | Dogs | Owned, sheltered and stray | Faecal samples (180) | Coprological methods | 1.7% | nd | Prevalence for each lifestyle was not discriminated | [94] |
|  |  | 2013 | Dogs | Owned | Faecal samples (302) | Macroscopic observation and coprological methods | 0.3% | nd | Positive case only detected during macroscopic observation | [95] |
|  |  | 2012 | nd | Soil: beaches, streets, and farms | Faecal samples (180) | Coprological methods | 13.9% | nd | na | [96] |
|  |  | 2010-11 | Cats | Owned and stray | GI contents (owned: 85; stray: 273) | Macroscopic and microscopic observation of parasites collected during necropsy | Owned – 29.4%; Stray – 29% | Owned: *Physaloptera praeputialis; T. canis; T. leonina.* Stray: *T. taeniformis*; *Taenia cati*;  *P. praeputialis*; *Ancylostoma tubaeforme*; *Moniliformis moniliformis* | na | [97] |
|  |  | 2008 | Dogs | Stray | GI contents (378) | Macroscopic and microscopic observation of parasites collected during necropsy | 44.9% | *Ancylostoma* sp.; *Toxocara* sp.; *Toxascaris* sp.; *Oslerus* sp.; *Spirocerca* sp.; *Taenia* sp.; *Physaloptera* sp. | na | [98] |
|  |  | 2002-04 | Cats | Owned | Adhesive tape from the perianal area and faecal samples (528) | Scotch tape or Graham’s test in the perianal area, and coprological methods | 0.76% | *Toxocara cati* | na | [99] |
|  |  | 1997-98 | Dogs | Stray | GI contents (120) | Macroscopic and microscopic observation of parasites collected during necropsy | 60% | nd | na | [100] |
|  |  | nd | Dogs | Owned | Faecal samples (130) | Coprological methods | 2.3% | *A. caninum*; *T. canis* | na | [101] |
|  | **USA** | 2017-20 | Dogs | Owned | GI contents and faecal samples (163) | Macroscopic and microscopic observation of parasites collected during necropsy, and coprological methods | 41.7% | nd | na | [102] |
|  |  | 2017-18 | Cats | Owned | Faecal samples (2586) | Coprological methods | 1.1% | nd | na | [103] |
|  |  | 2007-18 | Dogs | Owned | Faecal samples (7409) | Coprological methods | 0.84% | nd | na | [104] |
|  |  | 2017-18 | Cats | Sheltered | GI contents and faecal samples (56) | Macroscopic and microscopic observation of parasites collected during necropsy, and coprological methods | 29% | nd | Positive cases only detected during necropsy | [105] |
|  |  | 2015-16 | Cats | Stray | Faecal samples (846) | Macroscopic and microscopic observation of parasites collected during necropsy, and coprological methods | 4.5% | nd | na | [106] |
|  |  | 2010-11 | Cats | Sheltered | GI contents and faecal samples (116) | Macroscopic and microscopic observation of parasites collected during necropsy, and coprological methods | 34.5% | Fleas | na | [107] |
|  |  | 2006-10 | Cats | Sheltered | Faecal samples (1629) | Coprological methods | 1.1% | nd | na | [108] |
|  |  | 2009 | Dogs | Sheltered | Faecal samples (100) | Coprological methods | 2% | nd | na | [109] |
|  |  | 2000-07 | Dogs | Owned | Faecal samples (20991) | Macroscopic observation and coprological methods | 0.29% | nd | na | [110] |
|  |  | nd | Cats | Sheltered | Faecal samples (103) | Macroscopic and microscopic observation of parasites collected during necropsy, and coprological methods | 1% | nd | Positive cases only detected by coprological methods | [111] |
|  |  | nd | Dogs | Sheltered | GI contents and faecal samples (97) | Macroscopic and microscopic observation of parasites collected during necropsy, and coprological methods | 49.5% | *T. vulpis*; Fleas | na | [112] |
| **Central America** | **Cuba** | 2005-06 | Dogs | Stray | GI contents (461) | Macroscopic and microscopic observation of parasites collected during necropsy | 16.3% | nd | na | [113] |
| **South America** | **Brazil** | 2018-20 | na | Vegetables: street markets and supermarkets | Vegetables (60) | Sedimentation and flotation-centrifugation | 1.7% | nd | na | [114] |
|  |  | 2017-18 | Dogs; Cats | Owned | Faecal samples (dogs: 400; cats: 208) | Macroscopic observation and coprological methods | Dogs – 1.3%; Cats – 12.5% | Cats: Hookworms (cats) and fleas | Proglottids were detected in 54% of the positive cases | [115] |
|  |  | 2014-15 | Cats | Sheltered and stray | Faecal samples (sheltered: 91; stray: 172) | Coprological methods | Sheltered – 3.3%; Stray – 1.7% | Hookworms (both); *Cystoisospora felis* (stray) | na | [116] |
|  |  | 2011-15 | Cats | Owned | Faecal samples (60) | Coprological methods | 8.3% | *Ancylostoma* spp.; *Cystoisospora* spp.; *T. cati*; Fleas | Cats included in the study had GI clinical signs and positive copro-parasitological examination | [117] |
|  |  | 2012-13 | Dogs | Stray | GI contents (93) | Macroscopic and microscopic observation of parasites collected during necropsy | 61.3% | *A. caninum*; *Leishmania* sp. | na | [118] |
|  |  | 2010-11 | Cats | Stray | GI contents (146) | Macroscopic and microscopic observation of parasites collected during necropsy | 3.42% | nd | na | [119] |
|  |  | 2010 | Dogs | Stray | GI contents (155) | Macroscopic and microscopic observation of parasites collected during necropsy | 36.8% | nd | na | [120] |
|  |  | 2006-07 | Dogs | Owned | GI contents (7); Faecal samples (45) | Macroscopic and microscopic observation of parasites collected during necropsy; Coprological methods | Coprology – 8.9%; Necropsy – 57.1% | nd | na | [121] |
|  |  | 2007 | Cats | Stray | GI contents and faecal samples (51) | Macroscopic and microscopic observation of parasites collected during necropsy, and coprological methods | 21.6% | *Ancylostoma* spp.; *Toxocara* spp.; *Cystoisospora* spp.; *Cryptosporidium* | na | [122] |
|  |  | 2005 | Dogs | Stray | GI contents and faecal samples (46) | Macroscopic and microscopic observation of parasites collected during necropsy, and coprological methods | 45.7% | nd | Positive cases only detected during necropsy | [123] |
|  |  | 2004-05 | Dogs | Owned and stray | Faecal samples (owned: 125; stray: 129) | Coprological methods | Owned – 0%; Stray – 4.6% | nd | na | [124] |
|  |  | 2004-05 | Fleas | na | Fleas (1500 *C. felis felis* from 150 dogs) | Dissection of the flea with microscopical observation | 0.5% | Actinocephalidae | Positivity: cysticercoids detection | [125] |
|  |  | 2003-04 | Dogs | Owned | Faecal samples (401) | Coprological methods | 2.5% | nd | na | [126] |
|  |  | 2003-04 | Dogs | Stray | Faecal samples (158) | Coprological methods | 1.9% | nd | na | [127] |
|  |  | 1999-2000 | Dogs | Owned and stray | Faecal samples (owned: 119; stray: 152) | Coprological methods | 0.7% | nd | Prevalence for each lifestyle was not discriminated | [128] |
|  |  | nd | Dogs | Owned and sheltered | Faecal samples (owned: 57; sheltered: 336) | Macroscopic observation and coprological methods | Owned – 3.5%; Sheltered – 0.6% | Sheltered: *Toxocara* spp.; *Ancylostoma* spp. | na | [129] |
|  |  | nd | Dogs | Owned | Faecal samples (129) | Coprological methods | 2% | nd | na | [130] |
|  |  | nd | Dogs | Owned | Faecal samples (278) | Coprological methods | 1.1% | nd | Dogs included in the study were living with schoolchildren | [131] |
|  |  | nd | Cats | Owned | Faecal samples (173) | Coprological methods | 0.6% | nd | na | [132] |
|  |  | nd | Cats | Sheltered and stray | GI contents (sheltered: 36; stray: 99) | Macroscopic and microscopic observation of parasites collected during necropsy | Sheltered – 52.8%; Stray – 52.5% | nd | na | [133] |
|  | **Chile** | 2013 | Dogs | nd / soil: parks and public squares | Faecal samples (452) | Coprological methods | 2.6% | nd | na | [134] |
|  |  | 1996-2003 | Dogs; Cats | Owned | Faecal samples (dogs: 972, cats: 230) | Coprological methods | Dogs – 2.2%; Cats – 6.9% | nd | Animals included in the study had alterations in the faeces, or diarrhoea | [135] |
|  | **Colombia** | 2019 | Dogs; Cats  Humans | Owned / children | Faecal samples (dogs: 18; cats: 7; children: 23) | Coprological methods | Dogs – 20%; Cats and children – 0% | nd | na | [136] |
|  |  | 2018 | Dogs; Cats | nd | Faecal samples (dogs: 1111; cats: 203) | Coprological methods | Dogs – 1.3%; Cats – 2% | nd | Animals included in the study had diarrhoea | [137] |
|  |  | 2005-06 | na | Parks | Soil (1560) | Sedimentation and flotation | 0.1% | nd | na | [138] |
|  | **Argentina** | 2005-08 | nd | Soil: streets, parks, squares, and farms | Faecal samples (1944) | Coprological methods | 0.31% | nd | na | [139] |
| **Oceania** | **Australia** | 2014-15 | Dogs | Owned / soil: parks, beaches, and walking paths | Faecal samples (300) | Macroscopic observation and coprological methods | 0.3% | nd | na | [140] |
|  |  | 2003-06 | Dogs | Stray | GI contents (108) | Macroscopic and microscopic observation of parasites collected during necropsy | 0.9% | nd | na | [141] |

GI: Gastro-intestinal; nd: Not described; na: Not applicable. When coprological methods are mentioned, one or more of the following are included: faecal smear, flotation, and sedimentation.

**Table S2.** Case reports of *Dipylidium caninum* in humans, dogs, and cats (2000-2021)

| **Continent** | **Country** | **Sampling year** | **Species (origin / lifestyle; N)** | **Age** | **Clinical history** | **Haematological and biochemical alterations** | **Duration of symptoms** | **Previous diagnoses and treatments** | **Contact with dogs and/or cats** | **Diagnostic method** | **Treatment (dose)** | **Treatment outcome / secondary effects** | **Observations** | **Reference** |
| --- | --- | --- | --- | --- | --- | --- | --- | --- | --- | --- | --- | --- | --- | --- |
| **Asia** | **China** | nd | Human (1) | 17 months | Proglottids in the stool; Mild diarrhoea | High serum IgE level (255.8 IU/ml); Slightly reduced levels of haemoglobin and haematocrit | 1 month | Enterobiasis; Albendazole (200 mg) | Yes (pet dogs) | Microscopic examination of proglottids from stool | Praziquantel (1 dose, 25mg/kg); After 1 hour – 1 cup of Epsom salts: 10g of magnesium sulphate in 100ml warm water | Successful resolution / no side effects | na | [142] |
|  | **India** | 2011 | Dogs with fleas (Owned; 20) | nd | Proglottids in the faeces and anus, anorexia, weight loss, scratch of the perineal region against the wall | nd | nd | nd | na | Microscopic examination of parasites collected in the perineal/anal region | nd | nd | na | [143] |
|  |  | nd | Human (1) | 50 years | Proglottids in the stool; Lower abdominal pain; Occasionally diarrhoea and vomiting; Dry cough | Reduced haematocrit, low platelet count and elevation of erythrocyte sedimentation rate | 1 year | Albendazole | Yes (stray dogs) | Microscopic examination of proglottids from stool | Praziquantel (5 doses, 10mg/kg/day) | Successful resolution / nd | na | [144] |
|  |  | nd | Dogs (Owned; 3) | 1 year | Proglottids in the faeces; Diarrhoea; Scooting behaviour; Dullness; Poor hair coat, infested with fleas | nd | nd | nd | na | Microscopic examination of proglottids from faeces | Praziquantel (1 dose, 5mg/kg) | Successful resolution / nd | na | [145] |
|  |  | nd | Human (1) | 4 years | Proglottids in the stool; diarrhoea (for two days) | Reduced haematocrit | 6 months | Enterobiasis; Albendazole + antihistaminics | Yes (pet cats and stray dogs) | Microscopic examination of proglottids from stool | Praziquantel (1 dose, 10mg/kg) | Successful resolution / nd | na | [146] |
|  | **Israel** | nd | Dog (Owned; 1) | 6 weeks | Diarrhoea; Vomiting; Anorexia; Distended and painful abdomen; Intussusception; Tachycardia; Increased body temperature | Severe thrombocytopenia, anaemia, and hypoproteinaemia | nd | nd | na | Direct faecal smear | Praziquantel + Pyrantel + Febantel (1 tab/10 kg) | Euthanized due to complications in the clinical condition | The animal was co-infected with a variety of parasites, who had also contributed to these clinical signs and hematologic abnormalities | [147] |
| **Europe** | **Greece** | 2016-17 | Humans (10) | 7 months – 10 years | Chronic diarrhoea (2-3 weeks, n=4); Proglottids in the stool (n=3); Abdominal pain (n=2); Discomfort in defecation (n=1); Failure to thrive (n=1); Asymptomatic (n=1) | Severe leucocytosis, and eosinophilia (n=1) | nd | In one case: 4-year history of intestinal parasitic infections treated as enterobiasis – Also, after the *D. caninum* treatment, it was positive for *Enterobius vermicularis* | Yes (in 6/10 – dogs or cats) | Microscopic examination of proglottids or eggs from stool | Praziquantel (1 dose, 20mg/kg) (n=7); Praziquantel (2 doses, 20mg/kg with 2 weeks interval) (n=1); Praziquantel (2 doses, 20mg/kg with 1 month interval) (n=1); Praziquantel (2 doses, 20mg/kg with 2 weeks interval) and niclosamide (500mg, 2 months after praziquantel) (n=2) | Successful resolution / no side effects. Two cases were more challenging, with negative parasitology only 5-6 months after diagnosis | na | [148] |
|  |  | nd | Humans (3) | 7 months, and adult parents | Child: Proglottids in the stool; Distress in defecation. Parents: Asymptomatic | nd | 15 days | No | Only the parents (stray cat) | Microscopic examination of proglottids from stool, and direct smear of stools from parents | Praziquantel (100mg, 3 times daily, 1 day)  15 days later - second dose of praziquantel and niclosamide  (500mg) | No success – spontaneous resolution 2 months later / nd | na | [149] |
|  | **Poland** | nd | Human (1) | 2 years | Proglottids in the stool, underwear and in the bath water; Firstly asymptomatic; Abdominal pain; Sleep disorders and hyperactivity; Loss of appetite; Occasionally slimy stools | nd | 4 months | Pyrantel + Albendazole | Yes (pet dogs and cats, and stray cats) | Microscopic examination of proglottids from stool, direct smear of stools, and coprology | Praziquantel (1 dose, 10mg/kg) | Successful resolution / nd | Pets were also treated with praziquantel + fenbendazole, and with an ectoparasiticide | [150] |
|  | **Russia** | 1987-2017 | Humans (9) | 8 children: 11 months – 12 years. 1 adult: 27 years | Proglottids in the stool | nd | 3 months or less | Levamisole, mebendazole, albedazole, pyrantel | Yes (pet dogs and cats) | Microscopic examination of proglottids or strobila from stool | Praziquantel (1 dose, 15mg/kg) | Successful resolution / nd | Pets were also parasitised with *D. caninum* and had fleas. Then, were also treated with praziquantel (1 dose, 15 mg/kg) | [151] |
|  | **Spain** | nd | Human (1) | 11 months | Diarrhoea; Fever; Changes in stool appearance and frequency (green and liquid, 5 times per day); Enlarged and painful abdomen; Sadness and irritability | nd | 2 weeks | nd | Yes (dogs, occasionally) | Coprological methods | Praziquantel (1 dose, 150mg) | Successful resolution / nd | na | [152] |
|  | **Turkey** | nd | Human (1) | 26 years | Prolonged diarrhoea; Abdominal pain | nd | nd | Antimicrobial drugs to control the diarrhoea | No | Microscopic examination of the stool | Niclosamide (500 mg, 4 times a day for 1 day) | Successful resolution / nd | Pacient had an organ transplantand was being treated with prednisolone, tacrolimus, mycophenolic acid, pantoprazole, and acetylsalicylic acid | [153] |
| **North America** | **Mexico** | nd | Human (1) | 18 months | Proglottids in the stool (in two separate occasions) | nd | nd | Albendazole (400 mg) | No | Microscopic examination of proglottids and strobila from stool | Praziquantel (1 dose, 25mg/kg) and a mild laxative 1 hour later | Successful resolution / no side effects | Prior treatment with albendazole was not preceded by a diagnosis | [154] |
|  | **USA** | nd | Cat (Sheltered; 1) | 7 months | Proglottids in the perianal region | nd | nd | nd | na | Microscopic examination of proglottids and strobila collected during necropsy | na | na | The animal died during an ovariohysterectomy; Con-infections with various nematodes and cestodes | [155] |
|  |  | nd | Human (1) | 2 years | Proglottids in the stool; Perianal pruritus and mild dermatitis and linear excoriations in the perianal area; Sleep disorders and irritability | nd | 6 months | Suspicion of enterobiasis; Albendazole (2 doses) in all family members | Yes (pet dogs and one cat) | Presumptive diagnosis (based on clinical history and images of the parasites in the stool) | Praziquantel (1 dose, 10mg/kg) | Successful resolution / nd | Pet dogs had access to the exterior; Pets had no flea control | [156] |
|  |  | nd | Human (1) | 2 years | Proglottids in the stool; Mild perianal pruritus | nd | nd | nd | Yes (pet cats) | Microscopic examination of proglottids from stool | Praziquantel (1 dose) | Successful resolution / nd | Pet cats were also shedding similar worms and were empirically treated by a veterinarian | [157] |
|  |  | nd | Human (1) | 4 months | Proglottids in the stool (in two separate occasions); Abdominal pain; Irritability and agitation (especially at night) | Slight eosinophilia | nd | Diagnosed and treated 2 times as enterobiasis; Mebendazole | Yes (pet dog) | Microscopic examination of proglottids from stool | Praziquantel (1 dose) | Successful resolution / nd | Pet dog was dewormed two months before the symptoms in the child | [158] |
|  |  | nd | Human (1) | 2 years | Proglottids in the stool and perianal area; Mild epigastric pain | nd | 3 months | Diagnosed and treated 3 times as enterobiasis; Mebendazole (1 dose, 100 mg) | Yes (pet cat) | Microscopic examination of proglottids from stool | Praziquantel (1 dose, 150 mg) | Successful resolution / nd | Pet cat was shedding identical worms 3 months before the onset of symptoms in the child; Cat diagnosed by the veterinarian as tapeworm and successfully treated | [159] |
|  |  | nd | Human (1) | 6 months | Proglottids in the stool | nd | 3 months | nd | nd | Microscopic examination of proglottids from stool | nd | nd | Proglottids were previously confounded with vegetable seeds | [160] |
| **South America** | **Chile** | nd | Human (1) | 2 years | Proglottids in the stool; Bad hygienic conditions | nd | nd | nd | Yes (dogs and cat) | Microscopic examination of proglottids from stool | Praziquantel (1 dose) | Successful resolution / nd | Animals were infested with fleas and had no veterinary control | [161] |

nd: Not described; na: Not applicable

**References**

1. Elmonir W, Elaadli H, Amer A, El-Sharkawy H, Bessat M, Mahmoud SF, et al. Prevalence of intestinal parasitic infections and their associated risk factors among preschool and school children in Egypt. PLoS ONE. 2021;16:e0258037.

2. El-Seify MA, Aggour MG, Sultan K, Marey NM. Gastrointestinal helminths of stray cats in Alexandria, Egypt: a fecal examination survey study. Vet Parasitol Reg Stud Reports. 2017;8:104–6.

3. Khalafalla RE. A survey study on gastrointestinal parasites of stray cats in Northern Region of Nile Delta, Egypt. PLoS ONE. 2011;6:20283.

4. Gutema FD, Yohannes GW, Abdi RD, Abuna F, Ayana D, Waktole H, et al. *Dipylidium caninum* infection in dogs and humans in Bishoftu Town, Ethiopia. Diseases. 2020;9:1.

5. Johnson SAM, Gakuya DW, Mbuthia PG, Mande JD, Maingi N. Prevalence of gastrointestinal helminths and management practices for dogs in the Greater Accra region of Ghana. Heliyon. 2015;1:e00023.

6. Njuguna AN, Kagira JM, Karanja SM, Ngotho M, Mutharia L, Maina NW. Prevalence of *Toxoplasma gondii* and other gastrointestinal parasites in domestic cats from households in Thika region, Kenya. BioMed Res Int. 2017;2017:7615810.

7. Ezema KU, Malgwi SA, Zango MK, Kyari F, Tukur SM, Mohammed A, et al. Gastrointestinal parasites of dogs (*Canis familiaris*) in Maiduguri, Borno State, Northeastern Nigeria: risk factors and zoonotic implications for human health. Vet World. 2019;12:1150–3.

8. Okoye IC, Obiezue NR, Okorie CE, Ofoezie IE. Epidemiology of intestinal helminth parasites in stray dogs from markets in south-eastern Nigeria. J Helminthol. 2011;85:415–20.

9. Ugbomoiko US, Ariza L, Heukelbach J. Parasites of importance for human health in Nigerian dogs: high prevalence and limited knowledge of pet owners. BMC Vet Res. 2008;4:49.

10. Maikai BV, Umoh JU, Ajanusi OJ, Ajogi I. Public health implications of soil contaminated with helminth eggs in the metropolis of Kaduna, Nigeria. J Helminthol. 2008;82:113–8.

11. Sowemimo OA. The prevalence and intensity of gastrointestinal parasites of dogs in Ile-Ife, Nigeria. J Helminthol. 2009;83:27–31.

12. Sowemimo OA, Asaolu SO. Epidemiology of intestinal helminth parasites of dogs in Ibadan, Nigeria. J Helminthol. 2008;82:89–93.

13. Minnaar WN, Krecek RC, Fourie LJ. Helminths in dogs from a peri-urban resource-limited community in Free State Province, South Africa. Vet Parasitol. 2002;107:343–9.

14. Minnaar WN, Krecek RC. Helminths in dogs belonging to people in a resource-limited urban community in Gauteng, South Africa. Onderstepoort J Vet Res. 2001;68:111–7.

15. Sulieman Y, Zakaria MA, Pengsakul T. Prevalence of intestinal helminth parasites of stray dogs in Shendi area, Sudan. Ann Parasitol. 2020;66:115–8.

16. Lahmar S, Sarciron M-E, Rouiss M, Hammouda A, Youssfi M, Mensi M. Echinococcus granulosus and other intestinal helminths in semi-stray dogs in Tunisia: infection and re-infection rates. Tunis Med. 2008;86:279–86.

17. Bwalya EC, Nalubamba KS, Hankanga C, Namangala B. Prevalence of canine gastrointestinal helminths in urban Lusaka and rural Katete Districts of Zambia. Prev Vet Med. 2011;100:252–5.

18. Nonaka N, Nakamura S, Inoue T, Oku Y, Katakura K, Matsymoto J, et al. Coprological survey of alimentary tract parasites in dogs from Zambia and evaluation of a coproantigen assay for canine echinococcosis. Ann Trop Med Parasitol. 2011;105:521–30.

19. Yu Z, Ruan Y, Zhou M, Chen S, Zhang Y, Wang L, et al. Prevalence of intestinal parasites in companion dogs with diarrhea in Beijing, China, and genetic characteristics of *Giardia* and *Cryptosporidium* species. Parasitol Res. 2018;117:35–43.

20. Dai RS, Li ZY, Li F, Liu DX, Liu W, Liu GH, et al. Severe infection of adult dogs with helminths in Hunan Province, China poses significant public health concerns. Vet Parasitol. 2009;160:348–50.

21. Budke CM, Campos-Ponce M, Qian W, Torgerson PR. A canine purgation study and risk factor analysis for echinococcosis in a high endemic region of the Tibetan plateau. Vet Parasitol. 2005;127:43–9.

22. Moudgil AD, Mittra S, Agnihotri RK, Sharma D, Sen D. Prevalence of gastrointestinal parasites in dogs of Palampur, Himachal Pradesh. J Parasit Dis. 2016;40:227–9.

23. Qadir S, Dixit AK, Dixit P, Sharma RL. Intestinal helminths induce haematological changes in dogs from Jabalpur, India. J Helminthol. 2011;85:401–3.

24. Borthakur SK, Mukharjee SN. Gastrointestinal helminthes in stray cats (*Felis Catus*) from Aizawl, Mizoram, India. Southeast Asian J Trop Med Public Heal. 2011;42:255–8.

25. Rabbani IAR, Mareta FJ, Kusnoto, Hastutiek P, Lastuti NDR, Mufasirin, et al. Zoonotic and other gastrointestinal parasites in cats in Lumajang, East Java, Indonesia. Infect Dis Reports. 2020;12:8747.

26. Siyadatpanah A, Pagheh AS, Daryani A, Sarvi S, Hosseini SA, Norouzi R, et al. Parasitic helminth infections of dogs, wolves, foxes, and golden jackals in Mazandaran Province, North of Iran. Vet World. 2020;13:2643–8.

27. Siyadatpanah A, Gholami S, Daryani A, Sarvi S-D, Sharif M, Seguel M, et al. The prevalence of intestinal helminths in free-ranging canids of Mazandaran, Northern Iran. Iran J Parasitol. 2019;14:563–71.

28. Mirbadie SR, Nasab AN, Mohaghegh MA, Norouzi P, Mirzaii M, Spotin A. Molecular phylodiagnosis of *Echinococcus granulosus* sensu lato and *Taenia hydatigena* determined by mitochondrial Cox1 and SSU-rDNA markers in Iranian dogs: indicating the first record of pig strain (G7) in definitive host in the Middle East. Comp Immunol Microbiol Infect Dis. 2019;65:88–95.

29. Darabi E, Kia EB, Mohebali M, Mobedi I, Zahabiun F, Zarei Z, et al. Gastrointestinal helminthic parasites of stray cats (*Felis catus*) in Northwest Iran. Iran J Parasitol. 2021;16:418–25.

30. Mohaghegh MA, Vafaei MR, Ghomashlooyan M, Azami M, Falahati M, Azadi Y, et al. A wide diversity of zoonotic intestinal parasites in domestic and stray dogs in rural areas of Kermanshah province, Iran. Trop Biomed. 2018;35:82–90.

31. Yakhchali M, Hajipour N, Malekzadeh-Viayeh R, Esmaeilnejad B, Nemati-Haravani T, Fathollahzadeh M, et al. Gastrointestinal helminths and ectoparasites in the stray cats (Felidae: *Felis catus*) of Ahar Municipality, Northwestern Iran. Iran J Parasitol. 2017;12:298–304.

32. Hajipour N, Baran AI, Yakhchali M, Khojasteh SMB, Hesari FS, Esmaeilnejad B, et al. A survey study on gastrointestinal parasites of stray cats in Azarshahr, (East Azerbaijan province, Iran). J Parasit Dis. 2016;40:1255–60.

33. Emamapour SR, Borji H, Nagibi A. An epidemiological survey on intestinal helminths of stray dogs in Mashhad, North-east of Iran. J Parasit Dis. 2015;39:266–71.

34. Khademvatan S, Abdizadeh R, Rahim F, Hashemitabar M, Ghasemi M, Tavalla M. Stray cats gastrointestinal parasites and its association with public health in Ahvaz city, South Western of Iran. Jundishapur J Microbiol. 2014;7:e11079.

35. Beiromvand M, Akhlaghi L, Massom SHF, Meamar AR, Motevalian A, Oormazdi H, et al. Prevalence of zoonotic intestinal parasites in domestic and stray dogs in a rural area of Iran. Prev Vet Med. 2013;109:162–7.

36. Borji H, Razmi G, Ahmadi A, Karami H, Yaghfoori S, Abedi V. A survey on endoparasites and ectoparasites of stray cats from Mashhad (Iran) and association with risk factors. J Parasit Dis. 2011;35:202–6.

37. Nabavi R, Naeini KM, Zebardast N, Hashemi H. Epidemiological study of gastrointestinal helminthes of canids in Chaharmahal and Bakhtiari province of Iran. Iran J Parasitol. 2014;9:276–81.

38. Adinezadeh A, Kia EB, Mohebali M, Shojaee S, Rokni MB, Zarei Z, et al. Endoparasites of stray dogs in Mashhad, Khorasan Razavi Province, Northeast Iran with special reference to zoonotic parasites. Iran J Parasitol. 2013;8:459–66.

39. Zare-Bidaki M, Mobedi I, Ahari SS, Habibizadeh S, Naddaf SR, Siavashi MR. Prevalence of zoonotic intestinal helminths of canids in Mog-han Plain, Northwestern Iran. Iran J Parasitol. 2010;5:42–51.

40. Gholami S, Daryani A, Sharif M, Amouei A, Mobedi I. Seroepidemiological survey of helminthic parasites of stray dogs in Sari City, Northern Iran. Pakistan J Biol Sci. 2011;14:133–7.

41. Eslami A, Ranjbar-Bahadori S, Meshgi B, Dehghan M, Bokaie S. Helminth infections of stray dogs from Garmsar, Semnan Province, Central Iran. Iran J Parasitol. 2010;5:37–41.

42. Zibaei M, Sadjjadi M, Sarkari B. Prevalence of *Toxocara cati* and other intestinal helminths in stray cats in Shiraz, Iran. Trop Biomed. 2007;24:39–43.

43. Arbabi M, Hooshyar H. Gastrointestinal parasites of stray cats in Kashan, Iran. Trop Biomed. 2009;26:16–22.

44. Dalimi A, Sattari A, Motamedi G. A study on intestinal helminthes of dogs, foxes and jackals in the western part of Iran. Vet Parasitol. 2006;142:129–33.

45. El-Shehabi FS, Kamhawi SA, Schantz PM, Craig PS, Abdel-Hafez SK. Diagnosis of canine echinococcosis: comparison of coproantigen detection with necropsy in stray dogs and red foxes from Northern Jordan. Parasite. 2000;7:83–90.

46. Scholz T, Uhlířová M, Ditrich O. Helminth parasites of cats from the Vientiane Province, Laos, as indicators of the occurrence of causative agents of human parasitoses. Parasite. 2003;10:343–50.

47. Ngui R, Lee SC, Yap NJ, Tan TK, Aidil RM, Chua KH, et al. Gastrointestinal parasites in rural dogs and cats in Selangor and Pahang states in Peninsular Malaysia. Acta Parasitol. 2014;59:737–44.

48. Zain SNM, Sahimin N, Pal P, Lewis JW. Macroparasite communities in stray cat populations from urban cities in Peninsular Malaysia. Vet Parasitol. 2013;196:469–77.

49. Low VL, Prakash BK, Tan TK, Sofian-Azirun M, Anwar FHK, Vinnie-Siow WY, et al. Pathogens in ectoparasites from free-ranging animals: infection with *Rickettsia asembonensis* in ticks, and a potentially new species of *Dipylidium* in fleas and lice. Vet Parasitol. 2017;245:102–5.

50. Othman RA, Abuseir S. The prevalence of gastrointestinal parasites in native dogs in Palestine. Iran J Parasitol. 2021;16:435–42.

51. Jitsamai W, Khrutkham N, Hunprasit V, Chandrashekar R, Bowman D, Sukhumavasi W. Prevalence of endoparasitic and viral infections in client-owned cats in metropolitan Bangkok, Thailand, and the risk factors associated with feline hookworm infections. Vet Parasitol Reg Stud Reports. 2021;25:100584.

52. Rojekittikhun W, Chaisiri K, Mahittikorn A, Pubampen S, Sa-nguankiat S, Kusolsuk T, et al. Gastrointestinal parasites of dogs and cats in a refuge in Nakhon Nayok, Thailand. Southeast Asian J Trop Med Public Heal. 2014;45:31–9.

53. Inpankaew T, Traub R, Thompson R C, Sukthana Y. Canine parasitic zoonoses in Bangkok temples. Southeast Asian J Trop Med Public Heal. 2007;38:247–55.

54. Yong TS, Lee K-J, Shin MH, Yu HS, Suvonkulov U, Turycin BS, et al. Prevalence of intestinal helminth infections in dogs and two species of wild animals from Samarkand region of Uzbekistan. Korean J Parasitol. 2019;57:549–52.

55. Beugnet F, Labuschagne M, Fourie J, Jacques G, Farkas R, Cozma V, et al. Occurrence of *Dipylidium caninum* in fleas from client-owned cats and dogs in Europe using a new PCR detection assay. Vet Parasitol. 2014;205:300–6.

56. Knaus M, Kusi I, Rapti D, Xhaxhiu D, Winter R, Visser M, et al. Endoparasites of cats from the Tirana area and the first report on *Aelurostrongylus abstrusus* (Railliet, 1898) in Albania. Wien Klin Wochenschr. 2011;123 Suppl 1:31–5.

57. Xhaxhiu D, Kusi I, Rapti D, Kondi E, Postoli R, Rinaldi L, et al. Principal intestinal parasites of dogs in Tirana, Albania. Parasitol Res. 2011;108:341–53.

58. Diakou A, Sofroniou D, Cesare A, Kokkinos P, Traversa D. Occurrence and zoonotic potential of endoparasites in cats of Cyprus and a new distribution area for *Troglostrongylus brevior*. Parasitol Res. 2017;116:3429–35.

59. Dubná S, Langrová I, Nápravník J, Jankovská I, Vadlejch J, Pekár S, et al. The prevalence of intestinal parasites in dogs from Prague, rural areas, and shelters of the Czech Republic. Vet Parasitol. 2007;145:120–8.

60. Takeuchi-Storm N, Mejer H, Al-Sabi MNS, Olsen CS, Thamsborg SM, Enemark HL. Gastrointestinal parasites of cats in Denmark assessed by necropsy and concentration McMaster technique. Vet Parasitol. 2015;214:327–32.

61. Barutzki D, Schaper R. Results of parasitological examinations of faecal samples from cats and dogs in Germany between 2003 and 2010. Parasitol Res. 2011;109 Suppl 1:S45–60.

62. Barutzki D, Shaper R. Endoparasites in dogs and cats in Germany 1999-2002. Parasitol Res. 2003;90 Suppl 3:S148–50.

63. Symeonidou I, Gelasakis AI, Arsenopoulos K, Angelou A, Beugnet F, Papadopoulos E. Feline gastrointestinal parasitism in Greece: emergent zoonotic species and associated risk factors. Parasit Vectors. 2018;11:227.

64. Symeonidou I, Gelasakis A, Arsenopoulos KV, Schaper R, Papadopoulos E. Regression models to assess the risk factors of canine gastrointestinal parasitism. Vet Parasitol. 2017;248:54–61.

65. Diakou A, Cesare A, Accettura PM, Barros L, Iorio R, Paoletti B, et al. Intestinal parasites and vector-borne pathogens in stray and free-roaming cats living in continental and insular Greece. PLoS Negl Trop Dis. 2017;11:e0005335.

66. Kostopoulou D, Claerebout E, Arvanitis D, Ligda P, Voutzourakis N, Casaert S, et al. Abundance, zoonotic potential and risk factors of intestinal parasitism amongst dog and cat populations: the scenario of Crete, Greece. Parasit Vectors. 2017;10:43.

67. Papazahariadou M, Founta A, Papadopoulos E, Chliounakis S, Antoniadou-Sotiriadou K, Theodorides Y. Gastrointestinal parasites of shepherd and hunting dogs in the Serres Prefecture, Northern Greece. Vet Parasitol. 2007;148:170–3.

68. Fok E, Szatmári V, Busák K, Rozgonyi F. Prevalence of intestinal parasites in dogs in some urban and rural areas of hungary. Vet Q. 2001;23:96–8.

69. Traversa D, Cesare A, Simonato G, Cassini R, Merola C, Diakou A, et al. Zoonotic intestinal parasites and vector-borne pathogens in Italian shelter and kennel dogs. Comp Immunol Microbiol Infect Dis. 2017;51:69–75.

70. Veronesi F, Gazzonis AL, Napoli E, Brianti E, Santoro A, Zanzani SA, et al. Cross-sectional survey on *Tritrichomonas foetus* infection in Italian cats. Vet Parasitol Reg Stud Reports. 2016;6:14–9.

71. Sauda F, Malandrucco L, MacRì G, Scarpulla M, Liberato C, Terracciano G, et al. *Leishmania infantum*, *Dirofilaria* spp. and other endoparasite infections in kennel dogs in central Italy. Parasite. 2018;25:2.

72. Scaramozzino P, Carvelli A, Iacoponi F, Liberato C. Endoparasites in household and shelter dogs from Central Italy. Int J Vet Sci Med. 2018;6:45–7.

73. Zanzani SA, Cerbo AR, Gazzonis AL, Genchi M, Rinaldi L, Musella V, et al. Canine fecal contamination in a metropolitan area (Milan, North-Western Italy): prevalence of intestinal parasites and evaluation of health risks. Sci World J. 2014;2014:132361.

74. Riggio F, Mannella R, Ariti G, Perrucci S. Intestinal and lung parasites in owned dogs and cats from central Italy. Vet Parasitol. 2013;193:78–84.

75. Sherifi K, Rexhepi A, Hamidi A, Behluli B, Zessin K, Mathis A, et al. Detection of patent infections of *Echinococcus granulosus* (“sheep-strain”, G1) in naturally infected dogs in Kosovo. Berl Munch Tierarztl Wochenschr. 2011;124:518–21.

76. Sadowska N, Tomza-Marciniak A, Juszczak M. Soil contamination with geohelminths in children’s play areas in Szczecin, Poland. Ann Parasitol. 2019;65:65–70.

77. Felsmann MZ, Michalski MM, Felsmann M, Sokół R, Szarek J, Strzyżewska-Worotyńska E. Invasive forms of canine endoparasites as a potential threat to public health – A review and own studies. Ann Agric Environ Med. 2017;24:245–9.

78. Tylkowska A, Pilarczyk B, Gregorczyk A, Templin E. Gastrointestinal helminths of dogs in Western Pomerania, Poland. Wiad Parazytol. 2010;56:269–76.

79. Silva V, Silva J, Gonçalves M, Brandão C, Brito NV. Epidemiological survey on intestinal helminths of stray dogs in Guimarães, Portugal. J Parasit Dis. 2020;44:869–76.

80. Mateus TL, Castro A, Ribeiro JN, Vieira-Pinto M. Multiple zoonotic parasites identified in dog feces collected in Ponte de Lima, Portugal – A potential threat to human health. Int J Environ Res Public Health. 2014;11:9050–67.

81. Waap H, Gomes J, Nunes T. Parasite communities in stray cat populations from Lisbon, Portugal. J Helminthol. 2014;88:389–95.

82. Duarte A, Castro I, Fonseca IMP, Almeida V, Carvalho LMM, Meireles J, et al. Survey of infectious and parasitic diseases in stray cats at the Lisbon Metropolitan Area, Portugal. J Feline Med Surg. 2010;12:441–6.

83. Raičević JG, Pavlović IN, Galonja-Coghill TA. Canine intestinal parasites as a potential source of soil contamination in the public areas of Kruševac, Serbia. J Infect Dev Ctries. 2021;15:147–54.

84. Ilić T, Nišavić U, Gajić B, Nenadović K, Ristić M, Stanojević D, et al. Prevalence of intestinal parasites in dogs from public shelters in Serbia. Comp Immunol Microbiol Infect Dis. 2021;76:101653.

85. Millán J, Casanova JC. High prevalence of helminth parasites in feral cats in Majorca Island (Spain). Parasitol Res. 2009;106:183–8.

86. Rodríguez-Ponce E, González JF, de Felipe MC, Hernández JN, Jaber JR. Epidemiological survey of zoonotic helminths in feral cats in Gran Canaria island (Macaronesian archipelago-Spain). Acta Parasitol. 2016;61:443–50.

87. Martínez-Carrasco C, Berriatua E, Garijo M, Martínez J, Alonso FD, Ybáñez RR. Epidemiological study of non-systemic parasitism in dogs in southeast Mediterranean Spain assessed by coprological and post-mortem examination. Zoonoses Public Heal. 2007;54:195–203.

88. Martínez-Moreno FJ, Hernández S, López-Cobos E, Becerra C, Acosta I, Martínez-Moreno A. Estimation of canine intestinal parasites in Córdoba (Spain) and their risk to public health. Vet Parasitol. 2007;143:7–13.

89. Miró G, Mateo M, Montoya A, Vela E, Calonge R. Survey of intestinal parasites in stray dogs in the Madrid area and comparison of the efficacy of three anthelmintics in naturally infected dogs. Parasitol Res. 2007;100:317–20.

90. Miró G, Montoya A, Jiménez S, Frisuelos C, Mateo M, Fuentes I. Prevalence of antibodies to *Toxoplasma gondii* and intestinal parasites in stray, farm and household cats in Spain. Vet Parasitol. 2004;126:249–55.

91. Zottler EM, Bieri M, Basso W, Schnyder M. Intestinal parasites and lungworms in stray, shelter and privately owned cats of Switzerland. Parasitol Int. 2019;69:75–81.

92. Abdullah S, Helps C, Tasker S, Newbury H, Wall R. Pathogens in fleas collected from cats and dogs: distribution and prevalence in the UK. Parasit Vectors. 2019;12:71.

93. Trasviña-Muñoz E, López-Valencia G, Monge-Navarro FJ, Herrera-Ramírez JC, Haro P, Gómez-Gómez SD, et al. Detection of intestinal parasites in stray dogs from a farming and cattle region of northwestern Mexico. Pathogens. 2020;9:516.

94. De-La-Rosa-Arana J-L, Tapia-Romero R. Frequency of helminth eggs in faeces of puppies living in urban or rural environments of Mexico City. Iran J Parasitol. 2018;13:632–6.

95. Torres-Chablé OM, García-Herrera RA, Hernández-Hernández M, Peralta-Torres JA, Ojeda-Robertos NF, Blitvich BJ, et al. Prevalence of gastrointestinal parasites in domestic dogs in Tabasco, southeastern Mexico. Rev Bras Parasitol Vet. 2015;24:432–7.

96. Vélez-Hernández L, Reyes-Barrera K, Rojas-Almaráz D, Calderón-Oropeza MA, Cruz-Vázquez JK, Arcos-García JL. [Potential hazard of zoonotic parasites present in canine feces in Puerto Escondido, Oaxaca]. Salud Publica Mex. 2014;56:625–30. [Article in Spanish].

97. Cantó GJ, Guerrero RI, Olvera-Ramírez AM, Milián F, Mosqueda J, Aguilar-Tipacamú G. Prevalence of fleas and gastrointestinal parasites in free-roaming cats in central Mexico. PLoS ONE. 2013;8:e60744.

98. Cantó GJ, García MP, García A, Guerrero MJ, Mosqueda J. The prevalence and abundance of helminth parasites in stray dogs from the city of Queretaro in central Mexico. J Helminthol. 2011;85:263–9.

99. Cossío TLI, Luna ADM, Mejia MR, Ortega AF, Cárdenas RH, Núñez CR. Risk factors associated with cat parasites in a feline medical center. J Feline Med Surg Open Reports. 2021;7:1–9.

100. Eguía-Aguilar P, Cruz-Reyes A, Martínez-Maya JJ. Ecological analysis and description of the intestinal helminths present in dogs in Mexico City. Vet Parasitol. 2005;127:139–46.

101. Rodríguez-Vivas RI, Gutierrez-Ruiz E, Bolio-González ME, Ruiz-Piña H, Ortega-Pacheco A, Reyes-Novelo E, et al. An epidemiological study of intestinal parasites of dogs from Yucatan, Mexico, and their risk to public health. Vector-Borne Zoonotic Dis. 2011;11:1141–4.

102. Gruntmeir JM, Thompson NM, Long MT, Blagburn BL, Walden HDS. Detection of heartworm antigen without cross-reactivity to helminths and protozoa following heat treatment of canine serum. Parasit Vectors. 2021;14:71.

103. Nagamori Y, Payton ME, Looper E, Apple H, Johnson EM. Retrospective survey of parasitism identified in feces of client-owned cats in North America from 2007 through 2018. Vet Parasitol. 2020;277:109008.

104. Nagamori Y, Payton ME, Looper E, Apple H, Johnson EM. Retrospective survey of endoparasitism identified in feces of client-owned dogs in North America from 2007 through 2018. Vet Parasitol. 2020;282:109137.

105. Loftin CM, Donnett UB, Schneider LG, Varela-Stokes AS. Prevalence of endoparasites in northern Mississippi shelter cats. Vet Parasitol Reg Stud Reports. 2019;18:100322.

106. Nagamori Y, Payton ME, Duncan-Decocq R, Johnson EM. Fecal survey of parasites in free-roaming cats in northcentral Oklahoma, United States. Vet Parasitol Reg Stud Reports. 2018;14:50–3.

107. Little S, Adolph C, Downie K, Snider T, Reichard M. High prevalence of covert infection with gastrointestinal helminths in cats. J Am Anim Hosp Assoc. 2015;51:359–64.

108. Lucio-Forster A, Bowman DD. Prevalence of fecal-borne parasites detected by centrifugal flotation in feline samples from two shelters in upstate New York. J Feline Med Surg. 2011;13:300–3.

109. Tupler T, Levy JK, Sabshin SJ, Tucker SJ, Greiner EC, Leutenegger CM. Enteropathogens identified in dogs entering a Florida animal shelter with normal feces or diarrhea. J Am Vet Med Assoc. 2012;241:338–43.

110. Gates MC, Nolan TJ. Declines in canine endoparasite prevalence associated with the introduction of commercial heartworm and flea preventatives from 1984 to 2007. Vet Parasitol. 2014;204:265–8.

111. Hoggard KR, Jarriel DM, Bevelock TJ, Verocai GG. Prevalence survey of gastrointestinal and respiratory parasites of shelter cats in northeastern Georgia, USA. Vet Parasitol Reg Stud Reports. 2019;16:100270.

112. Adolph C, Barnett S, Beall M, Drake J, Elsemore D, Thomas J, et al. Diagnostic strategies to reveal covert infections with intestinal helminths in dogs. Vet Parasitol. 2017;247:108–12.

113. Merlo RH, Núñez FÁ, Durán LP. [Zoonotic potential of intestinal helminth infections in stray dogs from City of Havana]. Rev Cubana Med Trop. 2007;59:234–40. [Article in Spanish].

114. Rocha LFN, Rodrigues SS, Santos TB, Pereira MF, Rodrigues J. Detection of enteroparasites in foliar vegetables commercialized in street-and supermarkets in Aparecida de Goiânia, Goiás, Brazil. Braz J Biol. 2022;82:e245368.

115. Arruda IF, Ramos RCF, Barbosa AS, Abboud LCS, Reis IC, Millar PR, et al. Intestinal parasites and risk factors in dogs and cats from Rio de Janeiro, Brazil. Vet Parasitol Reg Stud Reports. 2021;24:100552.

116. Pereira PF, Barbosa AS, de Moura APP, Vasconcellos ML, Uchôa CMA, Bastos OMP, et al. Gastrointestinal parasites in stray and shelter cats in the municipality of Rio de Janeiro, Brazil. Rev Bras Parasitol Vet. 2017;26:383–8.

117. de Souza FB, Nakiri IM, de Lourenço NO, Silva GG, Paschoalini DR, Guimarães-Okamoto PTC, et al. Prevalence of intestinal endoparasites with zoonotic potential in domestic cats from Botucatu, SP, Brazil. Top Companion Anim Med. 2017;32:114–7.

118. Saldanha-Elias AM, Silva MA, Silva VO, Amorim SLA, Coutinho AR, Santos HA, et al. Prevalence of endoparasites in urban stray dogs from Brazil diagnosed with *Leishmania*, with potential for human zoonoses. Acta Parasitol. 2019;64:352–9.

119. Ramos DG, Scheremeta RG, Oliveira AC, Sinkoc AL, Pacheco RC. Survey of helminth parasites of cats from the metropolitan area of Cuiabá, Mato Grosso, Brazil. Rev Bras Parasitol Vet. 2013;22:201–6.

120. Heukelbach J, Frank R, Ariza L, Lopes ÍS, Silva AA, Borges AC, et al. High prevalence of intestinal infections and ectoparasites in dogs, Minas Gerais State (southeast Brazil). Parasitol Res. 2012;111:1913–21.

121. Santos JLC, Magalhães NB, Santos HA, Ribeiro RR, Guimarães MP. Parasites of domestic and wild canids in the region of Serra do Cipó National Park, Brazil. Rev Bras Parasitol Vet. 2012;21:270–7.

122. Coelho WMD, Amarante AFT, de Soutello RVG, Meireles MV, Bresciani KDS. [Occurrence of gastrointestinal parasites in fecal samples of cats in Andradina City, São Paulo]. Rev Bras Parasitol Vet. 2009;18:46–9. [Article in Portuguese].

123. Klimpel S, Heukelbach J, Pothmann D, Rückert S. Gastrointestinal and ectoparasites from urban stray dogs in Fortaleza (Brazil): high infection risk for humans? Parasitol Res. 2010;107:713–9.

124. Katagiri S, Oliveira-Sequeira TCG. Prevalence of dog intestinal parasites and risk perception of zoonotic infection by dog owners in São Paulo State, Brazil. Zoonoses Public Heal. 2008;55:406–13.

125. Avelar DM, Bussolotti AS, Ramos MCA, Linardi PM. Endosymbionts of *Ctenocephalides felis felis* (Siphonaptera: Pulicidae) obtained from dogs captured in Belo Horizonte, Minas Gerais, Brazil. J Invertebr Pathol. 2007;94:149–52.

126. Táparo CV, Perri SHV, Serrano ACM, Ishizaki MN, Costa TP, Amarante AFT, et al. [Comparison between coproparasitological techniques for the diagnosis of helminth eggs or protozoa oocysts in dogs]. Rev Bras Parasitol Vet. 2006;15:1–5. [Article in Portuguese].

127. Blazius R, Emerick S, Prophiro J, Romão P, Silva O. [Occurrence of protozoa and helminthes in faecal samples of stray dogs from Itapema City, Santa Catarina]. Rev Soc Bras Med Trop. 2005;38:73–4.

128. Oliveira-Sequeira TCG, Amarante AFT, Ferrari TB, Nunes LC. Prevalence of intestinal parasites in dogs from São Paulo State, Brazil. Vet Parasitol. 2002;103:19–27.

129. Ramos NV, Silva ML, Barreto MS, Barros LA, Mendes-De-Almeida F. Endoparasites of household and shelter cats in the city of Rio de Janeiro, Brazil. Rev Bras Parasitol Vet. 2020;29:1–15.

130. Curi NHA, Paschoal AMO, Massara RL, Santos HA, Guimarães MP, Passamani M, et al. Risk factors for gastrointestinal parasite infections of dogs living around protected areas of the Atlantic Forest: implications for human and wildlife health. Braz J Biol. 2017;77:388–95.

131. Oliveira-Arbex AP, David EB, Oliveira-Sequeira TCG, Katagiri S, Coradi ST, Guimarães S. Molecular identification of *Ancylostoma* species from dogs and an assessment of zoonotic risk in low-income households, São Paulo State, Brazil. J Helminthol. 2017;91:14–9.

132. Monteiro MFM, Ramos RAN, Calado AMC, Lima VFS, Ramos ICDN, Tenório RFL, et al. Gastrointestinal parasites of cats in Brazil: frequency and zoonotic risk. Rev Bras ParasitolVet. 2016;25:254–7.

133. Labarthe N, Serrão ML, Ferreira AMR, Almeida NKO, Guerrero J. A survey of gastrointestinal helminths in cats of the metropolitan region of Rio de Janeiro, Brazil. Vet Parasitol. 2004;123:133–9.

134. Luzio Á, Belmar P, Troncoso I, Luzio P, Jara A, Fernández Í. [Parasites of zoonotic importance in dog feces collected in parks and public squares of the city of Los Angeles, Bío-Bío, Chile]. Rev Chil Infectol. 2015;32:403–7. [Article in Spanish].

135. López J, Abarca K, Paredes P, Inzunza E. [Intestinal parasites in dogs and cats with gastrointestinal symptoms in Santiago, Chile]. Rev Méd Chile. 2006;134:193–200.

136. Peña-Quistial MG, Benavides-Montaño JA, Duque NJR, Benavides-Montaño GA. Prevalence and associated risk factors of intestinal parasites in rural high-mountain communities of the valle del Cauca –Colombia. PLoS Negl Trop Dis. 2020;14:e008734.

137. López-Arias Á, Villar D, López-Osorio S, Calle-Vélez D, Chaparro-Gutiérrez JJ. *Giardia* is the most prevalent parasitic infection in dogs and cats with diarrhea in the city of Medellín, Colombia. Vet Parasitol Reg Stud Reports. 2019;18:100335.

138. Polo-Terán LJ, Cortés-Vecino JA, Villamil-Jiménez LC, Prieto YE. [Zoonotic nematode contamination in recreational areas of Suba, Bogotá]. Rev salud pública. 2007;9:550–7. [Article in Spanish].

139. Soriano SV, Pierangeli NB, Roccia I, Bergagna HFJ, Lazzarini LE, Celescinco A, et al. A wide diversity of zoonotic intestinal parasites infects urban and rural dogs in Neuquén, Patagonia, Argentina. Vet Parasitol. 2010;167:81–5.

140. Gillespie S, Bradbury RS. A survey of intestinal parasites of domestic dogs in central Queensland. Trop Med Infect Dis. 2017;2:60.

141. Jenkins DJ, Allen L, Goullet M. Encroachment of *Echinococcus granulosus* into urban areas in eastern Queensland, Australia. Aust Vet J. 2008;86:294–300.

142. Jiang P, Zhang X, Liu RD, Wang ZQ, Cui J. A human case of zoonotic dog tapeworm, *Dipylidium caninum* (Eucestoda: Dilepidiidae), in China. Korean J Parasitol. 2017;55:61–4.

143. Wani ZA, Allaie IM, Shah BM, Raies A, Athar H, Junaid S. *Dipylidium caninum* infection in dogs infested with fleas. J Parasit Dis. 2015;39:73–5.

144. Meena S, Singh A, Kumar VP, Gupta R, Gupta P. *Dipylidium caninum*: first case in an adult female from uttarakhand and review of literature. Tropical Parasitology. 2020;10:153–7.

145. Saini VK, Gupta S, Kasondra A, Rakesh RL, Latchumikanthan A. Diagnosis and therapeutic management of *Dipylidium caninum* in dogs: a case report. J Parasit Dis. 2016;40:1426–8.

146. Narasimham MV, Panda P, Mohanty I, Sahu S, Padhi S, Dash M. *Dipylidium caninum* infection in a child: a rare case report. Indian J Med Microbiol. 2013;31:82–4.

147. Gal A, Harrus S, Arcoh I, Lavy E, Aizenberg I, Mekuzas-Yisaschar Y, et al. Coinfection with multiple tick-borne and intestinal parasites in a 6-week-old dog. CVJ. 2007;48:619–22.

148. Portokalidou S, Gkentzi D, Stamouli V, Varvarigou A, Marangos M, Spiliopoulou I, et al. *Dipylidium caninum* infection in children: clinical presentation and therapeutic challenges. Pediatr Infect Dis J. 2019;38:e157–9.

149. Xaplanteri P, Gkentzi D, Stamouli V, Kolonitsiou F, Anastassiou ED, Marangos M, et al. Rare worm in an infant’s nappy. Arch Dis Child. 2017;0:1.

150. Szwaja B, Romańsk L, Ząbczyk M. A case of *Dipylidium caninum* infection in a child from the southeastern Poland. Wiadomoœci Parazytol. 2011;57:175–8.

151. Bronstein AM, Fedyanina LV, Lukashev AN, Sergeev AR. Nine cases of human dipylidiasis in Moscow region during 1987 to 2017. Trop Biomed. 2020;37:194–200.

152. Rincon MJ, Gonzalez-Granado LI. [Pets and dipylidiasis]. An Pediatr. 2011;74:420. [Article in Spanish].

153. Sahin I, Köz S, Atambay M, Kayabas U, Piskin T, Unal B. A rare cause of diarrhea in a kidney transplant recipient: *Dipylidium caninum*. Transplant Proc. 2015;47:2243–4.

154. Cabello RR, Ruiz AC, Feregrino RR, Romero LC, Feregrino RR, Zavala JT. *Dipylidium caninum* infection. BMJ Case Reports. 2011;2011:bcr0720114510.

155. Lima JCMP, Piero FD. Severe concomitant *Physaloptera* sp., *Dirofilaria immitis*, *Toxocara cati*, *Dipylidium caninum*, *Ancylostoma* sp. and *Taenia taeniaeformis* infection in a cat. Pathogens. 2021;10:109.

156. Chong HF, Hammoud R, Chang ML. Presumptive *Dipylidium caninum* infection in a toddler. Case Reports Pediatr. 2020;2020:1–3.

157. Hogan CA, Schwenk H. *Dipylidium caninum* infection. N Engl J Med. 2019;380:e39.

158. Taylor T, Zitzmann MB. *Dipylidium caninum* in a 4-month old male. Clin Lab Sci. 2011;24:212–4.

159. Samkari A, Kiska DL, Riddell SW, Wilson K, Weiner LB, Domachowske JB. *Dipylidium caninum* mimicking recurrent *Enterobius vermicularis* (pinworm) infection. Clin Pediatr (Phila). 2008;47:397–9.

160. Molina CP, Ogburn J, Adegboyega P. Infection by *Dipylidium caninum* in an infant. Arch Pathol Lab Med. 2003;127:e157–9.

161. Neira OP, Jofré ML, Muñoz SN. [*Dipylidium caninum* infection in a 2 year old infant case report and literature review]. Rev Chil Infect. 2008;25:465–71. [Article in Spanish].
